# Multidimensional cerebellar computations for flexible kinematic control of movements

**DOI:** 10.1038/s41467-023-37981-0

**Published:** 2023-05-03

**Authors:** Akshay Markanday, Sungho Hong, Junya Inoue, Erik De Schutter, Peter Thier

**Affiliations:** 1 Hertie Institute for Clinical Brain Research, Eberhard Karls University Tübingen, Tübingen, Germany; 2 Computational Neuroscience Unit, Okinawa Institute of Science and Technology, Okinawa, Japan

**Keywords:** Neural encoding, Cerebellum, Saccades

## Abstract

Both the environment and our body keep changing dynamically. Hence, ensuring movement precision requires adaptation to multiple demands occurring simultaneously. Here we show that the cerebellum performs the necessary multi-dimensional computations for the flexible control of different movement parameters depending on the prevailing context. This conclusion is based on the identification of a manifold-like activity in both mossy fibers (MFs, network input) and Purkinje cells (PCs, output), recorded from monkeys performing a saccade task. Unlike MFs, the PC manifolds developed selective representations of individual movement parameters. Error feedback-driven climbing fiber input modulated the PC manifolds to predict specific, error type-dependent changes in subsequent actions. Furthermore, a feed-forward network model that simulated MF-to-PC transformations revealed that amplification and restructuring of the lesser variability in the MF activity is a pivotal circuit mechanism. Therefore, the flexible control of movements by the cerebellum crucially depends on its capacity for multi-dimensional computations.

## Abstract (web summary)

Moving precisely in natural environments requires adapting to multiple demands arising dynamically. Here, the authors show that the cerebellum’s capacity for multidimensional computations allows it to flexibly control multiple movement parameters guaranteeing movement precision.

## Introduction

Short-term motor learning is a specific variant of sensorimotor learning. It provides the ability to rapidly acquire a new control scheme that allows the motor system to cope with the demands of often unexpected or sudden changes in the external environment^1^. Not only external but also internal changes may require fast adjustments. For instance, the motor plant may change due to muscular fatigue slowing movements. Also, boredom and declining motivation, i.e., cognitive fatigue will reduce the speed of movements. If not too extensive, this slowing of movements—the decline of movement “vigor”—may not necessarily degrade endpoint precision as the speed reduction can be compensated by cranking up the overall movement duration, an adjustment of a distinct parameter that requires the cerebellum^2–4^. However, behavioral studies indicate that parametric control by the cerebellum, deployed to swiftly react to external and internal changes, is not confined to a single kinematic parameter like movement duration. Rather, work on goal-directed eye movements as models of cerebellum-based short-term motor learning has established that adaptation to external and internal changes involves adjustments of several kinematic parameters^2,5–11^.

How does the cerebellum coordinate the control of multiple kinematic parameters in order to ensure optimal movements? To answer this question, we should know at which stage of the cerebellar neural network the information on the various movement parameters and necessary adjustments is available and how they are transformed within the network. Previous studies on saccadic eye movements have emphasized the control of particular parameters like movement duration^12^ or velocity^13^ by the simple spike (SS) discharge of a population of cerebellar Purkinje cells (PCs)—the output currency of cerebellar cortex. Although it has been suggested that SS firing rate and spike time can simultaneously encode the velocity and timing of eye movement at the individual PC level^14^, ultimately unifying these divergent views at the population level is challenged by the large cell-to-cell variability in the discharge of cerebellar neurons. This problem is usually addressed by extensive averaging of all or categorized subsets of neurons in data^6,12,13,15,16^. However, averaging can lead to conclusions that are biased towards a particular parameter within a space of multiple encoded movement parameters and, in any case, it fails to detect information hidden in cell-to-cell variabilities. Unraveling such hidden information in neuronal populations is where recent studies of the neural dynamics of cortical motor regions have made remarkable progress^17–20^. One of the key ideas is that the apparent substantial heterogeneity—high dimensionality—of the responses of individual neurons can be explained by a combination of a smaller number of underlying patterns, i.e., a low-dimensional latent structure. This low-dimensional structure, referred to as the “*manifold*,” captures the essential properties residing inside the population discharge^20–22^ concealed by simple averaging across neurons without the risk of biased conclusions.

Hence, to address if and how the cerebellum accommodates the multifarious parametric requirements of short-term sensorimotor learning, here we identify the manifold structure of the activity of key input and output elements of the cerebellar cortical network, mossy fibers (MFs) and PCs, of nonhuman primates performing a fatigue-inducing repetitive saccade task entailing different kinematic changes. We report the multi-dimensional manifolds in the MF and PC-SS activity that simultaneously encode eye movement velocity and duration by their geometry and dynamics. We then proceed with considering the influence of climbing fibers, represented by the PC complex spike (CS) discharge on the PC manifolds, with a focus on its function of conveying information on performance error among others^23^. We show that CSs modulate PC manifolds in an error type-dependent manner that predicts complementary changes in subsequent eye movements by selectively controlling the individual movement parameters. Finally, we investigate the nature of the interaction between the input and output neurons and present evidence that the underlying network computation amplifies the relatively small variability in MF responses to transform them into representations of individual movement parameters, exhibited by PCs in an error-type-dependent manner. Our results demonstrate an enhanced computational capacity of PCs that provides the flexible control of more than one kinematic parameter, ensuring the precision of goal-directed movements.

## Results

### Velocity-duration adjustments during a fatigue-inducing repetitive saccade task

**Fig. 1:**
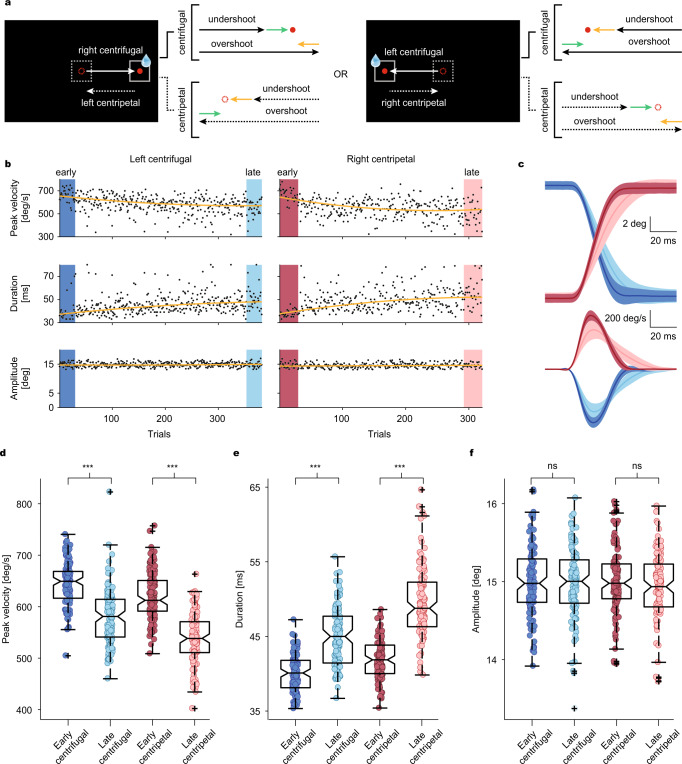
Repetitive saccade task induces a gradual decline in saccade velocity. **a** Behavioral task. Saccades were made repetitively, either in left or right directions. All center-out (centrifugal (CF), solid arrows) saccades were rewarded. Centripetal (CP) saccades (dashed arrows) were not rewarded. Both CF and CP saccades could lead to errors in leftward (orange arrows) or rightward (green arrows) directions. **b** Gradual decay of peak velocity (upper panels) in CF (left) and CP (right) saccades (CF: *p* = 1.12 × 10^−5^, *Z* = 4.4; CP: *p* = 1.02 × 10^−5^, *Z* = 4.4) is paralleled by an increase in duration (middle panels, CF: *p* = 1.82 × 10^−4^, *Z* = −3.7; CP: *p* = 1.9 × 10^−6^, *Z* = −4.8) to stabilize amplitudes (lower panels, CF: *p* = 0.89, *Z* = −0.1; CP: *p* = 0.95, *Z* = 0.1) within a single session. Comparison based on *n* = 30 early and late trials, respectively. Each dot represents data from a single trial. Trends in the data are highlighted by fitting second-order polynomial fits (dark yellow lines) to the data. All comparisons based on two-sided Wilcoxon signed-rank tests. **c** Comparison of horizontal eye position and velocity profiles of early (i.e., first 30 trials, CF: dark blue; CP: dark red) and late (i.e., last 30 trials, CF: light blue; CP: light red) trials chosen from the experimental session in **b**. Data are mean ± SD. **d**–**f** Population analysis of 117 behavioral sessions. Box plots showing overall reduction of peak velocity (CF: *p* = 6.51 × 10^−18^, *Z* = 8.6; CP: *p* = 8.81 × 10^−21^, *Z* = 9.3) in late trials (lighter colors) as compared to early (darker colors) ones which is compensated by the upregulation of saccade duration (CF: *p* = 1.31 × 10^−20^, *Z* = −9.3; CP: *p* = 8.81 × 10^−21^, *Z* = −9.3) during the late trials to maintain amplitude around 15 deg (CF: *p* = 0.57, *Z* = 0.6; CP: *p* = 0.01, *Z* = 2.5). Each data point corresponds to the mean value of the early (first 30, dark-colored circles) and late (last 30, light-colored circles) CF (blue circles) and CP saccades (red circles) of an individual session (*n* = 117 sessions). All comparisons based on two-sided Wilcoxon signed-rank tests. Significant differences are highlighted by asterisks. On each boxplot, center is median value, lower and upper edges of the box are 25th and 75th percentiles, respectively, whiskers extend to extreme values and outliers are marked as “+”.

We trained two monkeys to execute a long series of center-out visually guided saccades made towards two fixed target locations, left and right on the horizontal meridian (eccentricity: 15 deg), alternating between targets, in order to receive a water-based reward at the end of the movement (Fig. 1a, see Methods for details).

As exemplified in Fig. 1b–d, over the course of a session saccades exhibited a gradual decline in their peak velocity (PV), reflecting a general loss of motivation (“cognitive fatigue”), arguably due to the fast and repetitive nature of the task^4^ (Fig. 1b, up). This gradual drop in saccade velocities was compensated by a likewise gradual upregulation of saccade duration (Fig. 1b, middle) ensuring that endpoint accuracy was maintained (Fig. 1b, bottom) within an acceptable range of error (±2 deg around the target). Since inter-trial intervals were short (~100 ms), the monkeys had to execute rapid saccades back towards the fixation point (i.e., centripetal saccades) after every centrifugal saccade to get ready for the subsequent trial. Albeit not directly rewarded, the kinematic structure and the velocity-duration adjustments of centripetal saccades were very similar to those of centrifugal saccades (red and blue traces, Fig. 1b, c). The notion of a viable velocity-duration tradeoff suggested by the exemplary data received full support from a behavioral population analysis which was based on pooled saccades from all sessions in which we had recorded the responses of 117 MFs and a complementary dataset of saccades collected while recording from 151 PCs, the latter also the basis of Markanday et al.^23^ (Fig. 1d–f). Relative to the early trials, we observed an overall decrease of 9.9% in the median PV of late centrifugal and 12.1% decrease in late centripetal saccades (Fig. 1d), compensated by 12.2% and 16.5%, respectively, increases in median saccade duration (Fig. 1e), maintaining the required accuracy (Fig. 1f).

On top of these gradual changes, reflecting the consequences of the development of cognitive fatigue over many trials, we also observed a within-session, trial-to-trial variability in centrifugal and centripetal saccade endpoints (“motor noise”), which resulted from saccades randomly overshooting or undershooting the target (Fig. 1a, see schematic diagrams with green and yellow-colored arrows). Consequently, both saccade types could result in retinal errors in both directions that we could resort to when trying to estimate the preferred error direction of CSs fired by individual PCs as projected on the left–right axis.

### Mossy fiber discharge encodes saccade kinematics

**Fig. 2:**
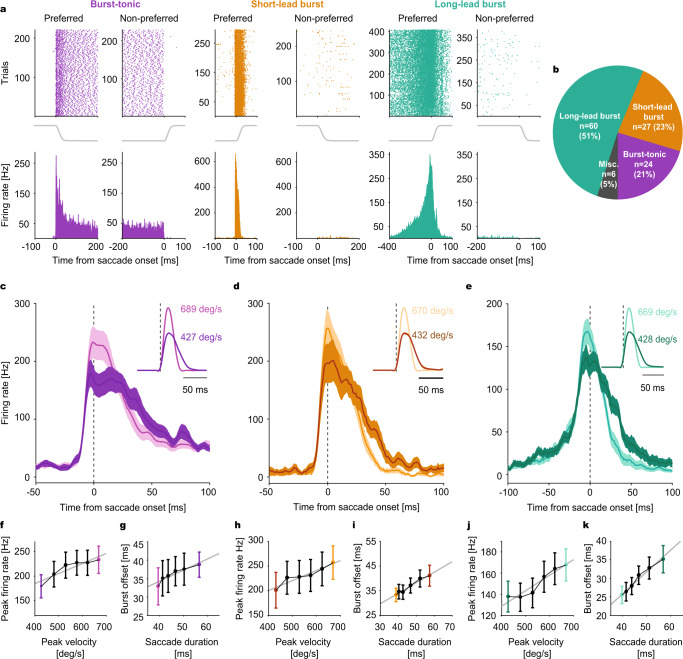
Encoding of saccade kinematics by mossy fibers (MFs). **a** Raster plots (up) and average firing histogram (bottom) of a representative burst-tonic (purple), short-lead burst (yellow) and long-lead burst (turquoise) MF unit. Solid gray lines between upper and lower panels are the mean horizontal eye position traces. Data are aligned to saccade onset. **b** Proportion of MF units in each category. **c**–**e** Population response of burst-tonic (purple), short-lead burst (yellow) and long-lead burst (turquoise) MFs to high and low velocity saccades (see insets for average velocity profiles), represented by lighter and darker shades, respectively. Solid lines represent the mean and the shaded regions are ±SEM. **f**, **h**, **j** Average peak firing rate as a linear function of saccade peak velocity (bin size = 50 deg/s) for each MF category. Linear regression parameters: Burst-tonic (**f**): *p* = 0.016, *R*^2^ = 0.83; Short-lead burst (**h**): *p* = 0.005, *R*^2^ = 0.9; Long-lead burst (**j**): *p* = 0.006, *R*^2^ = 0.9. **g**, **i**, **k** Average burst offset relative to saccade onset as a function of saccade duration (calculated from velocity bins) for each MF category. Linear regression parameters: Burst-tonic (**g**): *p* = 0.008, *R*^2^ = 0.88; Short-lead burst (**i**): *p* = 0.0005, *R*^2^ = 0.96; Long-lead burst (**k**): *p* = 0.0005, *R*^2^ = 0.97. Solid gray lines represent the linear regression fits. Light and dark-colored bins correspond to the high and low peak velocity bins, respectively, for which population responses in c, d and e are plotted for comparison. Data are mean ± SEM obtained from *n* = 24 burst-tonic, *n* = 60 long-lead burst and *n* = 27 short-lead burst units, respectively.

The saccade-related MFs, all recorded from the oculomotor vermis (OMV)^24,25^, could be broadly categorized into three main types —burst-tonic (BT), short-lead burst (SLB), and long-lead burst (LLB) units (Fig. 2b, see Methods for details), based on the timing of a burst-response component and the presence of subsequent tonic discharge. As demonstrated by an exemplary BT unit (Fig. 2a, left panels), a strong “burst” discharge for saccades made in the preferred leftward horizontal direction was followed by an elevated discharge rate (the tonic component), that persisted throughout the post-saccadic period and stopped only when the eyes began to move in the opposite (non-preferred) direction. Compared to the SLB and BT units that started to fire vigorously just a few milliseconds before saccade onset (Fig. 2a, middle; 9 ms in the example), the modulation onset of the LLB units occurred much earlier (Fig. 2a, right; ~330 ms in the example), reaching its maximum expression in a ramping manner. Independent of MF unit type, the discharge rate reached its peak during the saccade and stopped around the end of saccades, made into a unit´s preferred direction.

The discharge of MFs reflected the trial-to-trial changes in saccade kinematics. To demonstrate this relationship, we calculated the population responses for saccades in a unit´s preferred direction, separately for BT, SLB, and LLB MFs (*n* = 24, 27, and 60, respectively, Fig. 2b) and sorted them into bins of PV (bin size = 50 deg/s), ranging from low to high values (and corresponding changes in saccade duration). Comparing the MF population responses for the two extreme bins comprising the lowest and highest velocities, respectively, clearly showed that in all three MF groups (Fig. 2c–e), the peak firing rate was substantially larger for the high PV bin, associated with clearly shorter burst duration. Note that in all three classes of MFs, the peak discharge rate coincided with saccade onset and, moreover, that not only the saccade profiles but also the associated mean discharge profiles were clearly less skewed for the high PV bin. This was due to a shortening of the saccade deceleration phase and a parallel faster decay of the discharge following the discharge peak. In fact, the peak discharge rate grew linearly with PV over the full range of PV bins (Fig. 2f, h, j), whereas the time of burst offset (see Methods for details) linearly predicted the time of saccade offset (Fig. 2g, I, k). Even for the tiniest corrective microsaccades that occurred either during the fixation period or during the post-saccadic period after under- or overshooting saccades, we observed the same linear encoding of these kinematic parameters by the activity of the three MF types (Supplementary Fig. 1).

### Simple spikes of Purkinje cells encode saccade kinematics

**Fig. 3:**
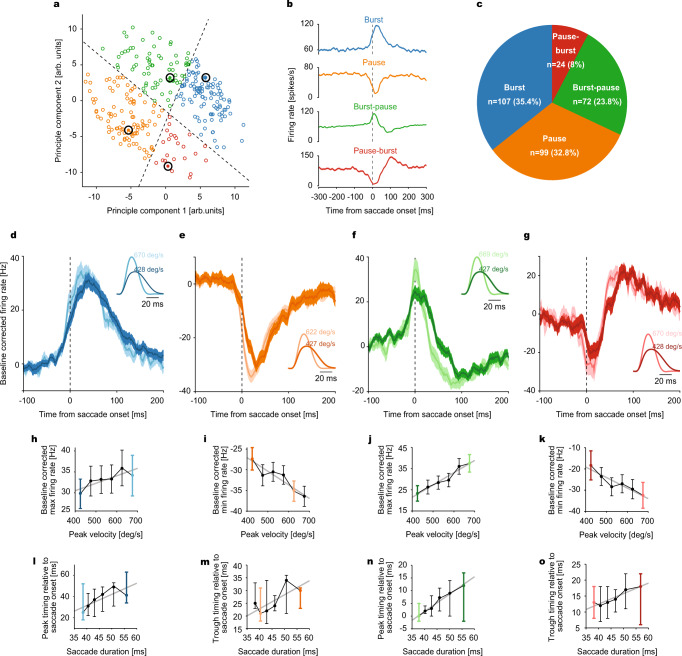
Classification of simple spike (SS) responses of Purkinje cells (PCs) into different categories and their encoding of saccade kinematics. **a** Scatter plot of the first two principal components of SS responses. Classification of PCs into four response categories: burst (blue), pause (orange), burst-pause (green) and pause-burst (red), separated by decision boundaries (dotted black lines). Each data point corresponds to a PC’s SS response in one of the two directions. **b** Saccade onset-aligned average SS responses of exemplary units taken from each category (large black circles in **a**). **c** The proportion of units in each category. **d**–**g** SS population response (baseline corrected, mean ± SEM) of all four categories to high and low velocity saccades (see insets for average velocity profiles), represented by lighter and darker shades, respectively. Data are aligned to saccade onset. **h**–**k** Baseline corrected, average maximum (**h**, **j**) and minimum (**i**, **k**) firing rates as a function of saccade peak velocity (bin size = 50 deg/s) for each category. Linear regression parameters: Burst (**h**): *n* = 107 units, *p* = 0.041, *R*^2^ = 0.69; Pause (**i**): *n* = 99 units, *p* = 0.0068, *R*^2^ = 0.87; Burst-pause (**j**): *n* = 72 units, *p* = 0.00081 *R*^2^ = 0.95; Pause-burst (**k**): *n* = 24 units, *p* = 0.0059, *R*^2^ = 0.88. **l**–**o** Average peak (for burst (*n* = 107) and burst-pause (*n* = 72) units; **l**, **n**) and trough (for pause (*n* = 99) and pause-burst (*n* = 24) units; **m**, **o**) timing relative to saccade onset as a function of saccade duration (calculated from velocity bins) for each PC category. Linear regression parameters: Burst (**l**): *p* = 0.065, *R*^2^ = 0.61; Pause (**m**): *p* = 0.087, *R*^2^ = 0.56; Burst-pause (**n**): *p* = 0.00015, *R*^2^ = 0.98; Pause-burst (**o**): *p* = 0.0059, *R*^2^ = 0.88. Solid gray lines represent the linear regression fits. Light and dark-colored bins correspond to the high and low peak velocity bins, respectively, for which population responses in **d**–**g** are plotted. Data are mean ± SEM.

We also recorded 151 OMV PCs and analyzed their SS responses. Whereas MFs exhibited bursting in their preferred saccade direction and little firing in the non-preferred direction, PC SS patterns for the two opposite directions—although often clearly different—did not follow a comparatively simple rule and exhibited a large variability in their response patterns (see Methods). Therefore, to characterize the different response patterns and their relation to saccade kinematics we considered SS responses for centripetal and centrifugal saccades separately. We classified them into four main categories—burst (*n* = 107), pause (*n* = 99), burst-pause (*n* = 72) and pause-burst types (*n* = 24), using linear discriminant analysis applied to the first two principal components accrued from a principle component analysis (PCA) of the discharge patterns (Fig. 3a, c, see Methods). The responses of typical “burst” and “pause” units were characterized by a saccade-related increase or decrease in firing rates respectively, whereas “burst-pause” and “pause-burst” units exhibited both types of changes, yet in opposite succession.

Pooling the responses of all SS units within each category, separately for the aforementioned PV bins, we obtained a clear linear relationship between the firing rate extremes (maximum discharge in units with burst component, minimal discharge in units with pause component) and eye velocity for all four SS categories (Fig. 3d–g and Fig. 3h–k). To capture saccade duration-related changes in SS firing, we relied on the timing of the first discharge rate extreme. As summarized in Fig. 3l–o, it shifted to later times in burst-pause and pause-burst units, while showing the same non-significant tendencies in the other two categories. Hence, one might conclude that the SS discharge of PCs in our data set encoded both movement velocity^13^ and duration^12^, albeit not as precisely as in the case of MFs.

### Identifying manifolds from pseudo-populations of MFs and PCs to unveil multi-dimensional coding of eye movements

However, there is a necessary caveat. Individual units were recorded in separate sessions. Hence the trial number varied between sessions. Moreover, the behavioral state of a monkey was hardly constant over sessions. Therefore, we must assume that such differences between individual sessions might have biased particular velocity bins. For instance, a session characterized by poor motivation would be expected to give rise to the emergence of low-velocity bins not found in sessions of higher motivation and, consequently, confounding estimates of the influence of PV on neuronal responses at the population level. In order to avoid confounded estimates of the kinematic dependencies of MF and SSs of PC units in our analysis, we resorted to a computational model that predicted the firing rate of individual MFs and PC-SSs based on a linear combination of a kinematics-independent component, namely the mean firing rate of a unit, and a PV- and/or duration-based modulation as added kinematics-dependent components (see Methods, Eq. 1). Finally, by combining the linear models of individual units, we obtained “pseudo-population” responses of MFs and PCs to which every unit contributed with an equal number of trials for any given PV bin as if all units had been recorded simultaneously during an experimental session^22^. A step-by-step illustration in Supplementary Fig. 3 shows the reconstruction of neuronal activities based on Eq. 1, using only PV as the kinematic-dependent parameter to compute the pseudo-population discharge as an estimate of the true population response (see also Methods for more details).

The population responses estimated by the pseudo-population model of MFs predicted the actual peak firing rate and duration of the population burst discharge with high accuracy (Supplementary Fig. 3e). On the other hand, the pseudo-population model for PC-SSs also predicted the same quantities significantly, but less well, especially the burst duration (Supplementary Fig. 3g). Note that the quality of the prediction did not improve substantially by considering both parameters (i.e., PV and duration) or only PV (see Supplementary Fig. 3e, g and Supplementary Methods). This is expected, since, for the maintenance of endpoint precision, a change of one kinematic parameter must be compensated by a coupled change of the other. Therefore, in most cases we used the PV-only model to probe the effects of the compensatory duration change correlated to PV change as in Figs. 2 and 3, except for when we investigated the effects of the uncorrelated changes in PV and duration by the PV and duration-based models, as discussed later. The relatively poor prediction provided by the pseudo-population of PC-SSs might be due to a much larger variability of the kinematics predictions of the individual models, reflected in higher standard errors of population averages of kinematics-independent and kinematics-dependent components (Supplementary Fig. 3c, bottom). A possible source of the high unit-to-unit variability could be the mixing of SS responses of individual PCs, each preferring a specific direction of retinal error. In fact, it has been shown that the conventional saccade-related SS population averages exhibit higher firing rates if the saccades considered are made in a direction that is opposite to the preferred direction of CSs, the latter the direction associated with the highest probability of observing CSs (CS-ON direction)^13^. Hence, could the performance of the PC-SS pseudo-population kinematics prediction be improved by grouping individual PC-SS responses into two pools that share the preference for error direction, i.e., left and right error, respectively? Indeed, reorganizing our PC data based on CS error-tuning, approximated by deciding whether left- or rightward errors evoked larger CS firing rates, led to a clearer saccade-related burst around the time of the saccade in the CS-OFF direction, whose peak clearly modulated with PV (Supplementary Fig. 4a, b), unlike for saccades made in the CS-ON direction (Supplementary Fig. 4c, d). However, despite qualitative differences in the CS-ON and OFF directions obtained by controlling for preferred error directions, the performance of the SS pseudo-population model in predicting the actual firing rates and burst duration did not improve compared to when information about CS-ON/-OFF directions was ignored (Supplementary Fig. 3e), suggesting that large heterogeneity in SS responses still prevailed. To quantify the difference in the kinematics encoding between MFs and PCs, we performed a PCA on PV-dependent components (reflecting PV encoding), similar to the PCA on the PV-independent mean firing rates shown in Fig. 3. We found that many more dimensions were required in the case of PCs (*d* = 10), compared to MFs (*d* = 4), to explain ~78% of the total variability in the encoding of PV by individual units (Supplementary Fig. 4e, f), supporting a large discrepancy in the heterogeneity of PV encoding by MFs and PCs.

**Fig. 4:**
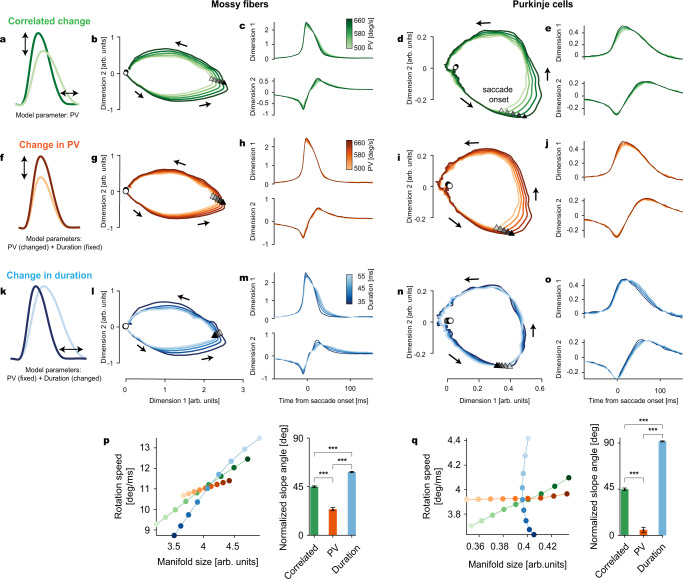
Manifolds identified in MF and PC-SS activity perform multi-dimensional encoding of eye movements. **a** Correlated changes in peak velocity (PV) and duration when PV is used as the only control parameter. **b** 2D plot of the first two dimensions in the MF manifold. Triangles and circles mark the saccade onsets and 250 ms before saccade onsets, respectively. Arrows show the direction of rotation. **c** The first two dimensions in **b**, plotted in time. **d**, **e** Same as **b**, **c** for PCs. **f** Isolated changes in saccade PV with the duration kept constant. **g**, **h** Isolated PV-dependent changes in the MF manifold computed from the rate models parametrized by PV but with fixed duration. **i**, **j** Same as **g**, **h** for PCs. **k** Isolated changes in saccade duration with constant PV. **l**–**o** Same as **g**, **h** and **i**, **j** but for duration change. **p** Left: MF manifold size versus rotation speed along the MF manifold varying with the correlated (green; **a**) and independent (orange and blue; **f**, **k**) change of PV and duration. Colors are as the color bars in **c**, **h**, **m**. Right: slope angle of the lines in left. In computing the angles, the *x*- and *y*-coordinates (manifold size and rotation speed) are normalized by the standard deviation of the correlated change case. T-val (Correlated, PV) = 17.97; ***: *p* = 1.27 × 10^−35^, T-val (PV, Duration) = −30.37; ***: *p* = 2.44 × 10^−57^, T-val (Correlated vs Duration) = −19.18; ***: *p* = 4.46 × 10^−38^. **q** Same as **p** for PCs. T-val (Correlated, PV) = 19.75; *p* = 5.26 × 10^−44^, T-val (PV, Duration) = −47.18; *p* = 1.36 × 10^−92^, T-val (Correlated vs Duration) = −48.13; ***: *p* = 8.24 × 10^−94^. *p* Values are from one-sided Student’s *t* tests. Data are jackknife mean ± SEM from *n* = 117 MFs and *n* = 151 PCs.

To mitigate the impact of this apparent large cell-to-cell variability, we resorted to dimensionality reduced representations of the pseudo-population responses of MFs and PC-SSs, each given as a linear combination of components, the first one reflecting the mean kinematics-independent firing rate and the second, kinematics-dependent discharge contribution reflecting discharge deviations due to fluctuations in movement kinematics. To this end, we used the following approach. First, we ran a PCA on the kinematics-independent component to identify the number of dimensions explaining a major chunk of the total cell-to-cell variability of the mean firing rates. However, there is a risk that these dimensions may change due to spontaneous trial-by-trial changes in PV or duration. Therefore, we resorted to matrix perturbation theory where we applied minor disturbances in PV or duration resembling trial-to-trial changes in our data and show that there are no significant changes in these dimensions as reflected by the eigenvalues of individual dimensions (Supplementary Fig. 5i). Given that the PCA-derived dimensionality is stable enough, we finally computed how the result of the first step would change relative to changes in movement parameters derived from the same dimensions of the kinematics-dependent component (see Methods, Eq. 2). This provided a good prediction of the movement parameter-dependent pseudo-population discharge (see Supplementary Methods for mathematical details). For MFs, the first step found two dimensions that explained 87.6% of the total cell-to-cell variability (Supplementary Fig. 5b). The second step found that the first dimension seemed to represent a burst modulation (Supplementary Fig. 5c, top and Fig. 4c, top), similar to the population average firing whose burst size and duration were modulated by PV. The second dimension (Supplementary Fig. 5c, bottom and Fig. 4c, bottom) represented the sustained responses during the pre- and post-saccadic period, reminiscent of the long-lead and tonic component of the LLB and BT MF types, respectively. Here, those components had opposite signs (rather than being all positive in the average firing rates), indicating an anti-correlation between the pre- and post-burst responses. However, in PCs, capturing 92.3% of the total cell-to-cell variability required four dimensions, where the first two dimensions represented simple monophasic (i.e., bursting or pausing) and biphasic (burst-pause or pause-burst) firing patterns, respectively, whereas the remaining two dimensions exhibited more complex features (Supplementary Fig. 5f, g and Fig. 4e).

We then plotted the first two of these reduced dimensions as a function of each other (“2D manifolds”, or in short “manifolds”), both for MFs and PCs for different values of PV. While both MF and PC manifolds appeared as limit cycle-like rotating trajectories, they exhibited crucial differences from each other (Supplementary Fig. 5d, h and Fig. 4b, d). For example, unlike the MF manifolds that were characterized by an overall PV-related increase in their size almost symmetrically around the saccade onsets, the PC-SS manifolds based on the first two dimensions showed no significant changes before saccade onsets, as depicted by the strong overlapping of the manifolds (Fig. 4a–e). However, the PC manifolds for the third and fourth dimensions showed clear differences already before saccade onsets (Supplementary Fig. 5h, bottom). Therefore, PC manifolds based on different dimensions can selectively encode specific phases of a movement, preparation and execution, in the same manner as the “null-space” in cortical manifolds for the preparation of reaching arm movements^18,19^, while the MF manifolds lacked this information suitable to control specific movement phases.

Furthermore, PC manifolds also carried a more disentangled representation of the two saccade parameters, PV and duration, compared to MFs. To arrive at this conclusion, we estimated the PV and duration-based models of MFs and PC-SSs. Since PV and duration in our data are highly, yet not perfectly correlated, these parameters had a residual variability, apart from the variability explained by the velocity-duration tradeoff suggesting complementary changes in PV and duration. In fact, the natural end-point variability we observed in saccades is a consequence of these residual variabilities appearing as slight deviations from the values predicted by the velocity-duration tradeoff. To capture the residual variability, we independently manipulated these two kinematic parameters (varying one while keeping the other fixed) and then tried to identify concomitant changes in MF manifolds. In fact, a change in PV (Fig. 4f) modulated the manifold size (i.e., geometry), as well as the time-dependence (i.e., rotating neural dynamics). While the manifold size can be interpreted as the maximal size of firing rate modulation, the rotation speed represents how rapidly the rate is modulated (Fig. 4g, h, see Methods for calculating manifold size and rotation-speed). Therefore, in terms of behavior, an increase in the manifold size can be linked to an increase in the PV of saccades, whereas a higher rotation speed of the manifold would suggest shorter saccade duration. Manipulating the saccade duration (Fig. 4k) also modified the MF manifolds (Fig. 4l, m) in a manner quite similar to the one resulting from correlated changes in PV and duration (Fig. 4a) where the perfect velocity-duration tradeoff is assumed. In contrast, PV (Fig. 4i, j) and saccade duration (Fig. 4n, o) varied the PC manifold size and rotating neural dynamics almost independently. These effects are summarized by the curves obtained by plotting the average rotation speed as a function of manifold size and their corresponding slope angles (Fig. 4p, q). Compared to the correlated change case (green in Fig. 4p, q), the curves with steeper slopes (or larger angles) indicate a bias towards the rotation speed (related to duration) adjustment, whereas smaller slope angles suggest stronger changes in the manifold size (related to PV). Therefore, while the slope angles were all comparable in the case of MFs (Fig. 4p), the PV and duration variation resulted in nearly orthogonal curves in the case of PCs, indicating almost completely decorrelated encoding of the two kinematic parameters (Fig. 4q).

Centrifugal saccades could be either leftwards or rightwards, but, notably, we found that our results did not depend on saccade direction. To test the potential influence of saccade direction on MF and PC manifolds, we performed the same analysis on MF and PC data separately for leftward and rightward saccades. For MFs, the left and right groups showed comparable results (Supplementary Fig. 6a, b) as suggested by high canonical correlations yielded by a canonical correlation analysis (CCA)^21,26^ (Supplementary Fig. 6c). In the PC case, the size of the manifold was much larger for saccades in the rightward direction as compared to leftward saccades (Supplementary Fig. 6d, e). Since around 80% of the recorded PCs had their CS-OFF in the rightward direction, the direction-dependent differences in the size of these manifolds are not surprising and only confirm the gain-field encoding of SSs^13^ (Supplementary Fig. 6g–i). Nevertheless, the shape of these manifolds was highly similar (Supplementary Fig. 6f). Therefore, MFs and PCs had qualitatively identical manifold structures regardless of the eye movement direction.

### PC manifolds reveal the structure of plasticity triggered by sensorimotor errors

In the prevailing theory for cerebellum-dependent sensorimotor learning, the climbing fiber-driven CSs convey motor error-related information to prompt parametric adjustments for correcting future motor behavior, thereby acting as “teacher signals”^27–29^. Therefore, motor learning has been attributed to these CSs, serving as a proxy of sensory feedback on motor errors that, when coincident with the parallel fiber inputs, modify the PC output by inducing a long-term depression (LTD) at the parallel fiber-PC synapses^30^.

**Fig. 5:**
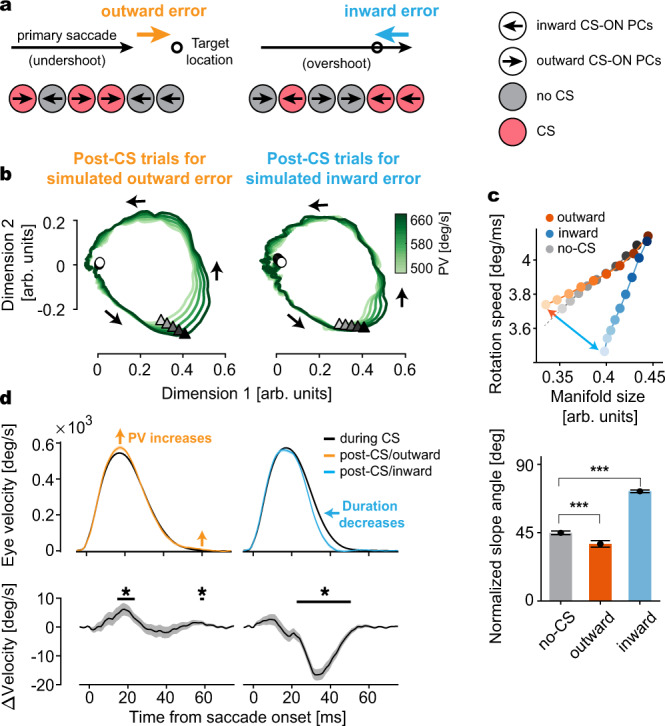
Complex spike (CS)-driven plasticity of PC manifolds is error-state dependent and predicts eye movement change. **a** Two different types of eye movement errors and CS firing in PCs encoding the errors. Left: An undershooting eye movement causes an outward error (orange) and CS firing in a population of PCs with the same CS-ON direction (red) but not in the other, CS-OFF PCs (gray). Right: Same as Left for an overshooting saccade causing an inward error (blue). **b** Left: PC manifolds reflecting the combined influence of simulated outward error-encoding CS firing pattern on a particular trial, obtained by combining CS trials of CS-ON PCs (red circles) and no-CS trials of CS-OFF PCs (gray circles), on subsequent trials. Note that, in simulated error trials we assume that CS-ON PCs reported an error by firing a CS during 50–140 ms after saccade offset, whereas CS-OFF PCs reported the same error by not firing a CS, irrespective of the actual presence of an error. Right: same as Left, but for the inward error. **c** Top: manifold size versus rotation speed after the outward (orange) and inward (blue) error-encoding CS trials, and after no-CS trials (gray). Color bar gradient represents PV from 500 deg/s (brightest) to 660 deg/s (darkest). Bottom: comparison of normalized slope angles for each condition. Data are jackknife mean ± SEM from *n* = 151 PCs. T-val (No-CS, Outward) = 4.11; ***: *p* = 3.18 × 10^−5^, T-val (Outward, Inward) = −20.76; ***: *p* = 2.20 × 10^−46^, T-val (No-CS, Inward) = −25.33; ***: *p* = 1.54 × 10^−56^. *p* Values are from one-sided Student’s *t* tests. **d** Top: average saccade velocity profiles in the CS (black) and post-CS trials (colored) for the simulated outward (left) and inward (right) errors. For highlighting the differences in velocity profiles, colored lines represent the cumulative effect of five CSs. Bottom: average eye velocity change from the CS to post-CS trials. Data are mean ± SEM. *p < 0.05 (two-sided Student’s *t* test).

To understand how the occurrence of CS impacts the multi-dimensional encoding of eye movements, we investigated how CSs fired during the post-saccadic period of 50–140 ms in the *n*^th^ trial (“CS trial”), reflecting retinal errors arising from natural end-point variability in saccades^23^, modulated the PC-SS manifolds of the subsequent, *n* + 1^th^ trials (“Post-CS trial”). In our paradigm, errors occurred mainly when the primary saccade undershot (outward error) or overshot (inward error) the target location (Fig. 5a). Depending on the direction of the primary saccade, these inward and outward errors could occur in both left and right directions (Fig. 1a). Therefore, depending on the CS-ON direction of individual PCs, the inward and outward errors will elicit CSs with high probability in those PCs whose CS-ON directions are aligned with the error vector (Fig. 5a, red circles), as compared to those cases in which the CS-ON direction and the error vector do not match^13,15,23,31,32^ (Fig. 5a, gray circles, also see Supplementary Fig. 7a). In other words, for any retinal error in a particular trial, there will always be a subpopulation of PCs whose CS-ON direction matches the error vector, leading to CS trials, and in others not, leading to “No-CS” trials. For a given error in the *n*^th^ trial, we looked at its influence on the entire population of PCs in our data set and the consequences for the SS manifolds of the *n* + 1^th^ trials, rather than restricting our analysis to only CS-ON units (see Supplementary Fig. 7b), assuming that the behavior is based on the concerted action of both subpopulations. To this end, we combined trials following CS-trials from the pool of CS-ON PCs (i.e., post-CS trials) and “No-CS” trials from CS-OFF PCs (“Post-no CS trials”), separately for outward (Fig. 5b, left) and inward errors (Fig. 5b, right). Importantly, we included all “CS trials” from CS-ON PCs (regardless of whether the actual error occurred or not) assuming that every CS in the error time window of 50–140 ms after the saccade was fired to report an error (referred to as simulated error trials in Fig. 5, see Supplementary Fig. 7a for a detailed illustration).

We found that CS firing associated with inward and outward errors modified the resulting PC-SS manifolds, based on PV as the kinematic-dependent parameter, differently (Fig. 5c, top). Relative to the “Post-no-CS” trials, the normalized slope angle, capturing changes in PV-dependent manifold size relative to the rotation speed, profoundly increased in the post-inward error trials but decreased, albeit only slightly, for post-outward error trials (Fig. 5c, bottom). Could it be that this result may be influenced by the actual error direction, rather than error type? Our analysis comparing inward and outward errors made in the same direction revealed that the PC-SS manifolds of subsequent trials maintained their specificity for inward and outward errors, even if their vectors pointed in the same direction (Supplementary Fig. 7c–e). Given that the PC manifold size and speed of the latent dynamics encode PV and saccade duration almost independently (Fig. 4q), this result suggested that CSs associated with inward and outward errors, potentially engaging the same population of PCs, tuned the population firing more towards duration coding in post-inward error trials (more compensatory duration change given PV) and PV coding in post-outward error trials (more compensatory PV change given duration).

Therefore, one would expect to see a reduction in subsequent saccade’s duration if a CS signal reporting an inward error, caused by an overshooting saccade in the previous trial, was present. On the other hand, in case of an outward error (i.e., undershooting saccade), CSs should trigger an increase in the PV of the next trial to reduce endpoint error. Indeed, this is what we found. When comparing the movement velocity of saccades accompanied by a CS to post-CS saccades, we observed that outward errors (undershooting) were corrected mainly through increasing the PV of the subsequent saccade with a slight increase in the velocity at the end of the saccade (Fig. 5d, left). In contrast, inward error-encoding CSs prompted a significant decrease in the duration of the subsequent saccade, reflected by the narrowing of its velocity profile (Fig. 5d, right).

Eye movement error-triggered CS firing is not restricted to our chosen post-saccadic error period of 50–140 ms^15,23^. Hence, how would CSs that fired at other time points influence the SS manifolds? We tested the influence of relatively late CSs during 140–250 ms from saccade offset^23^. We found that those late CSs also modified the SS manifolds of post-simulated inward and outward error trials, albeit differently from the earlier ones (Supplementary Fig. 8). These late CSs modified the PC-SS manifold in a way that prompted adjustments only in the duration of the subsequent saccades, for both inward and outward errors. Therefore, the CS timing may play an additional role in the selective control of kinematic parameters.

### Linear feed-forward network model shows high-dimensional transformations by the cerebellar cortex

We demonstrated that, despite the similar limit-cycle-like properties of MF and PC manifolds, there were crucial differences in their encoding of kinematic parameters, especially for PCs which also exhibited a large heterogeneity in their kinematics-independent and kinematics-dependent components. The climbing fiber-driven CSs clearly explain some of the differences between the two (Fig. 5 and Supplementary Fig. 6). However, additional inputs to PCs arriving from interneurons may also play a significant role.

**Fig. 6:**
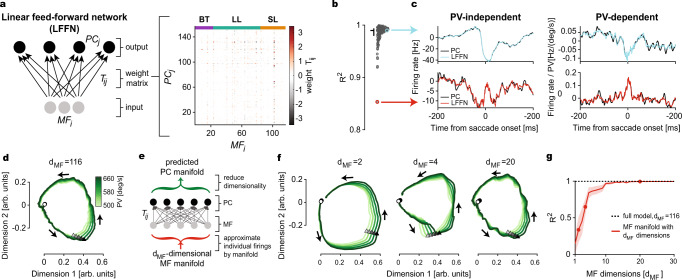
Linear feed-forward network (LFFN) model from MFs to PCs. **a** Left: schematic diagram showing LFFN for MF-to-PC firing rate transformation. Right: weight matrix computed from the data. Horizontal color bars on the top correspond to the three MF categories (BT burst-tonic, LL long-lead burst, SL short-lead burst). **b** Goodness of fit (*R*^2^) for individual PCs. The horizontal bar represents the median and the vertical bar extends from the first to third quantile, respectively. Colored circles correspond to examples shown in **c**. **c** Firing rates of example PCs (black, top and bottom) and LFFN predictions (color). The baselines are subtracted in the PV-independent component (left). **d** LFFN prediction of PC manifolds in Fig. 4d using all dimensions in MF firing. **e** Schematic diagram of the LFFN model showing steps involved (from bottom to top) in the prediction of PC manifolds from *d*_MF_-dimensional MF manifolds. **f** Examples of the predicted PC manifold from **e** when MF manifold dimension is *d*_MF_ = 2 (left), 4 (middle), and 20 (right). **g** Goodness of fit for the predicted PC manifold to the data versus the input MF manifold dimensions *d*_MF_. Dots represent examples in **f**. Data are mean ± SEM.

The role of input from climbing fibers and interneurons notwithstanding, we found that already a linear feed-forward network (LFFN) from MFs to PCs^33^ (Fig. 6a), not considering the aforesaid elements, was able to predict the kinematics-independent and kinematics-dependent activity components of all individual PCs with high fidelity (*R*^2^ = 0.984 ± 0.018, mean ± SD) (Fig. 6b, c), allowing us to successfully reproduce the PC-SS manifolds from the MF activity (Fig. 6d; see also Supplementary Fig. 9a–c). But how is it possible that already a simple linear transformation can explain the many differences between MF and PC-SS manifolds? This paradox led us to examine how many dimensions of MF (*d*_MF_) firing are necessary to make good predictions of the PC manifold.

We addressed this question by two approaches, both leading to the conclusion that the number of dimensions that need to be considered while trying to account for the properties of MF activity is definitely much smaller than the maximum number of dimensions, *d*_MF_ = 116 (corresponding to the number of MFs used in this analysis), but significantly higher than two or four, the dimensionalities capturing a major chunk of cell-to-cell variability in MFs and PCs, respectively (Supplementary Fig. 5a–h). In the first approach, we first created the *d*_MF_-dimensional pseudo-population firing rate model of MFs (Fig. 6e, gray circles) using the *d*_MF_-dimensional MF manifold (red arrow), then generated the prediction of individual PC-SS firings using the LFFN (black circles), and finally identified the predicted PC-SS manifold (green arrow). The PC-SS manifold (in the first two dimensions) was relatively poorly predicted (*R*^2^ < 0.9) when *d*_MF_ < 9 (Fig. 6f, g). In the second approach (Supplementary Fig. 9d), we directly tested whether the prediction of individual PC firings requires high dimensional components in MF firings by another LFFN model, where MFs and PCs communicate through a dimensionally reduced submanifold, called the “communication subspace”^34^. This model also showed that a good prediction of individual PC responses requires a high-dimensional (*d* > 15) communication subspace (Supplementary Fig. 9d,e). Note that the dimensions higher than four (i.e., *d* > 4) explain only 4.3% of the total MF-to-MF variance together due to rapid decay (∝1/*d*^3.23^) in the explained variance (Supplementary Fig. 5b). Therefore, the properties of multi-dimensional control of movements by PC-SS manifolds emerge from transformations by the cerebellar cortical circuit that amplifies those small variabilities in MF inputs.

## Discussion

The present study demonstrates the presence of multi-dimensional manifolds, latent in the activities of the cerebellar input and output, MFs and PCs respectively, and how their geometric and dynamic features encode key kinematic eye movement parameters (see Supplementary Fig. 10 for summary). Climbing fiber-driven CSs, signaling error-related information to PCs, modify the PC manifolds, differentially depending not only on the direction of error but also the type of error, which predicts how the subsequent eye movements are corrected. Finally, we show that the cerebellar cortical circuit amplifies seemingly insignificant variabilities in the MF activity to generate highly selective PC outputs.

The fast and repetitive nature of our paradigm induced cognitive fatigue, a gradual decline in the speed of saccades, which was compensated by duration upregulation^8^. However, on top of fatigue, we also observed natural trial-to-trial changes in the saccade velocity requiring rapid duration adjustments in order to guarantee endpoint precision. Therefore, the same velocity-duration trade-off mechanism that maintained movement accuracy across hundreds of trials within a session also ensured reduced endpoint variability (motor noise) on a trial-to-trial basis. The residual motor noise led to tiny, albeit specific error types, directed inward and outward respectively, depending on whether the eye movements were too large or fell short relative to the target location.

Depending on the firing pattern of individual MFs and SSs of PC units, we could broadly classify them into different categories by using strict statistical criteria to compute population averages of each category^13,25,35^. Yet, in our analysis, these units appeared continuous in their distribution (Supplementary Fig. 2) rather than forming discrete clusters, due to a large cell-to-cell variability exceeding between-category distances. Therefore, one may question the reliability of the classify-and-average approach in testing the encoding of specific kinematic parameters as it may be prone to the risk of sampling bias. This problem gets even worse if one additionally considers the large between-session variability in eye movements also influencing the firing rates of individual units. To avoid exactly these biases, we estimated the firing rates of all individual units, based on a firing rate model that varies linearly with key kinematic parameters, to obtain a “pseudo-population” of MFs and PC-SSs.

This new approach allowed us to identify multi-dimensional, limit cycle-like manifolds of neuronal activity from dimensionality reduction of the pseudo-population responses capturing a significant proportion of cell-to-cell variability^22^. Moreover, these low-dimensional components exhibited a mixture of different features observed in the firing patterns of individual MF and PC-SS responses, thereby capturing specific interactions between individual units to generate activity patterns relevant for selective kinematic control of movements. In the MF case, this cell-to-cell variability could be attributed to different inputs from a variety of premotor nuclei in the brain stem. For instance, the SLB MFs may represent the activity pattern of paramedian pontine reticular formation (PPRF) short-lead burst neurons^36^. The origin of the LLB types seems less clear. Candidate sources may include as well the PPRF but also the nucleus reticularis tegmenti pontis (NRTP) and the dorsal pontine nuclei^25,36–43^. These various nuclei provide the precise information on the kinematics of macro- and micro-saccades to MFs needed (Fig. 2 and Supplementary Fig. 1). And in the case of PCs, it might be the concerted impact of the granule cells-parallel fibers^44^ together with interneurons^45^ that accounts for the large heterogeneity in PC responses reflected by their larger number of low-dimensional components (Supplementary Fig. 4e, f).

PC discharge, the output of the cerebellar cortex, is only a few synapses away from the final stage motor neurons. Therefore, moving up the cerebellar circuitry, one would expect the PC signals to be far more refined and informative about the movements than the signals at earlier stages, e.g., at the level of MF afferents. At first glance, our results from the population analysis seemed to contradict this expectation as the MF pseudo-population exhibits a much more precise encoding of relevant kinematic parameters while the kinematics description provided by PC-SS pseudo-population responses is comparatively sloppy as a consequence of the large heterogeneity of individual firing patterns. However, a very different perspective is opened if one resorts to the low-dimensional pseudo-population manifolds that reveal the hidden dynamics of PC-SS activity for the flexible control of key movement parameters like velocity and duration in a movement phase specific manner. Furthermore, the PC manifolds carried significantly more disentangled representations of movements than the MF manifolds. Unlike MFs, the PC-SS manifolds exhibited distinct geometric and dynamical properties related to the two specific kinematic parameters, velocity, and duration. This conspicuous difference between the MF and the PC-SS manifolds indicates a highly nontrivial transformation by the network.

What is the nature of this transformation? Notably, a simple LFFN model^33^ simulating the MF-to-PC pathway could accurately explain the MF-to-PC transformation at the firing rate and manifold level, but only if the high dimensional components in the MF inputs, representing a tiny fraction (<5%) of the total MF-to-MF variability, were preserved. This result suggests that a disentangled movement encoding at the PC level emerges through substantial amplification of those seemingly insignificant variabilities in MF responses by the cerebellar network. Highly correlated activity, resulting in an apparently small dimensionality, has been widely observed in work on the cerebellar input layer^46–48^ (but see also ref. ^44^). We found the same in our MF data, but our analysis together with the PC data suggests that enhancing small input variabilities is a fundamental information processing principle of the cerebellar network. This expansion of variability may allow PCs to extract as much information as possible during the movement so it can be used optimally when errors occur. Therefore, the cerebellar circuit can predict the dynamical, trial-to-trial deviations from the forward model for the average eye movement. This property is important for the cerebellum’s function and role in dynamic, online movement control. Furthermore, together with the finding that serial single-unit recordings are sufficient to generate reliable MF and PC manifolds, the prediction power of the LFFN model implies that MFs should use asynchronous firing rate coding.

PCs are also influenced by the direct climbing fiber pathway, imparting plastic changes in their activity via CSs. Indeed, we found that CSs modulated the geometry and dynamics of the PC-SS manifolds on a trial-to-trial basis, in an error-type dependent manner, predicting selective post-CS parametric adjustments of eye movements. The forced error-based short-term saccadic adaptation is similarly error-type dependent^6^, which supports that PCs, by duration coding, control movements flexibly in response to external and internal (fatigue) changes^8^. On the other hand, recent studies have demonstrated the effects of CS-driven plasticity on the movement velocity, thereby emphasizing velocity-coding by PCs^13,15^. We demonstrate that those two mechanisms coexist and can be interwoven to exhibit complex forms of population-level plasticity. Notably, other properties of CS firing, such as the time of occurrence of these spikes may also govern the selective control of kinematic parameters by PCs. We found that CSs that fired relatively earlier during the post-saccadic retinal error period modified SS manifolds such that they predicted a PV change in post-outward error trials. However, when CSs fired later, the same errors were compensated by only a duration change. This result may seem perplexing as different periods of CS activity suggest different kinematic adjustments and yet, the motor system selects only one optimal behavior for the next trials. However, given that CSs signals carry a multiplexed code of behaviorally relevant information^23^, the different dimensions in PC firing may utilize these valuable CS signals, staggered in time, by integrating all the multiplexed information to make context-dependent, appropriate behavioral adjustments. Therefore, the multidimensional nature of cerebellar computations is necessary for the flexible, context-dependent control of movements and their rapid adaptation.

Our findings provide a novel outlook on the existing theories of cerebellar function in sensorimotor control. Particularly, the so-called internal model hypothesis proposed that the cerebellum operates as a predictive circuit, implementing a control-theoretic internal model that estimates an outcome of a controlled system to an input^49,50^. With the pioneering use of the LFFN analysis (Fig. 6), an earlier study supported this theory by showing that the final output of the cerebellum, the deep cerebellar nuclei, can predict the external network input provided by MFs^33^. Our results significantly advance this concept by elucidating how the cerebellum can process the efference copy inputs bearing multi-dimensional dynamics, which can arise potentially in similar ways to the motor/premotor and collicular regions related to other motor behavior where earlier studies showed the existence of neural manifolds^17–19,21,51^. Furthermore, this study highlights the cerebellar function in processing and learning to modify the trial-to-trial variability in movement and neural activity. This capability is crucial in rapid, online motor control, but has remained elusive due to studies usually analyzing only trial-averaged body and neural dynamics. Based on the observations reported in this study, we conclude that the cerebellar neural circuit performs optimal computations for fast and flexibly varying sensorimotor control, indispensable in natural environments.

## Methods

### Animals, preparation, and surgical procedures

Two healthy male rhesus macaques (*Macaca mulatta*; monkey K and monkey E, age: 10 years and 8 years, respectively), purchased from the German Primate Center in Göttingen, were used for the purpose of this study. All data presented in this study were collected from these two animals using procedures that strictly adhered to the rules defined by the German as well as the European law and guidelines that were approved by the local authority (Regierungspräsidium Tübingen, veterinary license N7/18 and N4/14) and National Institutes of Health’s *Guide for the Care and Use of Laboratory Animals*. All training, experimental and surgical procedures were supervised by the veterinary service of Tübingen University.

As a first step, the animals were subjected to chair training which began in the animal facility where animals were encouraged to voluntarily enter a customized mobile chair for the first few weeks following which they were transported to the experimental area where they were gradually acclimatized to the new environment. To proceed with experimental training, it was necessary to painlessly immobilize the head in order to record eye movements reliably. Therefore, once the animals felt fully comfortable in the experimental setups, the first major surgical procedure of installing the foundations of cranial implants was performed. During this procedure, the scalp was cut open and these foundations, made from titanium, were fixed to the skull using titanium bone screws. The scalp was then closed with the help of sutures under which the foundations were allowed to rest and stabilize for at least 3-4 months to ensure their durability and also full recovery of the animals. After this period, the second surgical procedure was performed in which the scalp was opened just enough to allow a titanium-based hexagonal tube-shaped head-post to be attached to the base of the implanted head-holder. Since this procedure was rather quick, the surgery was also accompanied by implantation of magnetic scleral search coils^52,53^ to record high-precision eye movements. After 2–3 weeks of recovery, monkeys were trained further on the behavioral task until their performance was accurate enough to consider neural recordings. To this end, the final surgical procedure was performed in which the upper part of the cylindrical titanium recording chamber (tilting backward by an angle of 30° with respect to the frontal plane, right above the midline of the cerebellum) was attached to the already implanted chamber foundation. A small area of the skull within the confines of the chamber was removed to allow electrode access to our region of interest, the oculomotor vermis (OMV, lobules VIC/VIIA). The position and orientation of the chamber were carefully planned and confirmed based on pre-and post-surgical MRI, respectively. All surgical procedures were performed under aseptic conditions using general anesthesia in which all vital physiological parameters (blood pressure, body temperature, heart rate, pO_2_ and pCO_2_) were closely monitored^54^. After surgery, analgesics (buprenorphine) were delivered to ensure painless recovery which was monitored using regular ethograms under the strict supervision of animal caretakers and veterinarians.

### Experimental set-up and behavioral task

All experiments were performed inside a dark room where monkeys, with their heads fixed, were seated comfortably in a primate chair placed at 38 cm in front of a CRT monitor such that their body axis was aligned to the center of the monitor. All neural and behavioral data presented in this study were collected during a simple to-and-fro saccade task in which monkeys were asked to rapidly shift their eye gaze repeatedly in order to follow a jumping target that appeared in two fixed locations along the horizontal axis on the monitor in an alternating manner (Fig. 1a). Before the beginning of each trial, the fixation target (a red dot of diameter 0.2 deg) appeared at the center of the monitor with an invisible fixation window of size 2 × 2 deg centered on it. Only if the monkeys moved their gaze within the fixation window the trial was initiated. This was followed by a short fixation period ranging from 400 to 600 ms from trial onset after which the fixation target vanished and, at the same time, another target (with the same properties as the fixation target) appeared at a new horizontal location, giving the impression that the target “jumped” centrifugally (Fig. 1a, solid arrows), i.e., from the center of the screen to this new location. The size (=15 deg) and the direction (left or right) of the target jump were kept constant within a session. Every target jump served as a “go-cue” which prompted the monkey to execute a saccade towards the new target location within the 2 x 2 deg fixation window centered on it, in order to receive an instantaneous reward (water drops) marking the end of a trial. The peripheral target disappeared approximately 700–900 ms relative to the go-cue, immediately after which the central fixation dot reappeared indicating the beginning of the next centrifugal trial. To proceed with the next trial, the monkey made a saccade from the peripheral target back to the central location (i.e., centripetal saccade, see dashed arrows in Fig. 1a). In other words, the appearance of the central fixation dot served as a go-cue for centripetal saccades, although these saccades were not rewarded. Depending on the motivation of the monkeys to perform the task, as well as the duration for which a PC could be kept well isolated, the number of trials varied in each session (median = 307 trials) with each trial lasting for 1200 ms. While the fatigue-inducing fast and repetitive nature of the paradigm allowed us to capture both trial-by-trial and gradually declining changes in the peak velocity of centrifugal and centripetal saccades, the natural endpoint variability in saccades, on the other hand, observed as over- or undershoots resulting in inward (Fig. 1a, see yellow arrows) or outward errors (Fig. 1a, see green arrows), allowed us to measure the CS’s preferred and anti-preferred direction of error for an individual PC. All experimental parameters were designed and controlled using in-house Linux-based software, NREC (http://nrec.neurologie.uni-tuebingen.de).

### Electrophysiological recordings, identification of Purkinje cells and mossy fibers in the oculomotor vermis

All electrophysiological recordings of PCs (*n* = 151) and mossy fibers (*n* = 117) from the OMV were performed using glass-coated tungsten microelectrodes (impedance: 1–2 MΩ), manufactured by Alpha Omega Engineering, Nazareth, Israel. To target the OMV, as predicted by the MRI scans, the position of electrodes along the rostrocaudal (i.e, *Y*-axis) and lateral (i.e, *X*-axis) axis were manually adjusted with the help of a custom-made microdrive, temporarily mounted on the recording chamber during each experimental session. The depth of the electrode was controlled using a modular multi-electrode manipulator (Electrode Positioning System and Multi-Channel Processor, Alpha Omega Engineering). The exact location of the OMV was confirmed based on careful inspection of online audio-visual feedback of the electrode signals, reflecting multi-unit granule cells activity, that exhibited strong modulations in response to fast eye movements.

For PC recordings, extracellular potentials sampled at 25 KHz were high (300 Hz–3 KHz) and low (30–400 Hz) band-pass filtered to obtain action potentials and LFP signals, respectively. Individual PC units were identified based on the presence of two types of action potential signals, high-frequency simple spikes (SSs) and low-frequency complex spikes (CSs), the latter characterized by a polyphasic wave morphology in the action potential trace paralleled by large deflections in the LFP signals. The fact that both signals originate from the same unit was confirmed online by the suppression of SS discharge for 10–20 ms when aligned to the occurrence of a CS^55–57^. Although the final characterization of CSs was based on an offline neural networks approach^58^, we relied on the performance of Alpha Omega Engineering´s Multi Spikes Detector for detecting SSs online. Note that we did not analyze the LFP signals since they are only weakly correlated to the firing rate code for movement kinematics and seem to represent other types of information^14^, which are beyond the scope of this paper.

In order to record from mossy fibers (MFs) in the granular layer, we adjusted the upper cut-off frequency of the high band-pass filter to 5 KHz while keeping the lower cut-off frequency the same as 300 Hz. The identification of MFs was based on their strong directionally selective response to saccades, firing up to several hundred spikes per second in the preferred direction and seldomly in the opposite direction. Unlike the relatively longer duration SSs (mean duration: 1.5 ms), MF units exhibited much shorter duration (mean duration: 0.6 ms), mostly mono- and biphasic shaped waveforms while occasionally exhibiting a negative after-wave^16,24,25,59,60^. Additionally, MFs exhibited a wide range of inter-spike intervals^16^ (mean ± sd: 82.7 ± 86 ms) as compared to those of PC SSs (mean ± sd: 19.5 ± 2.6 ms).

### Classification of mossy fiber responses

Unlike the bidirectional SS discharge of PCs, well-isolated MF units exhibited a strong and clear preference for saccades made in one of the two horizontal directions. This property allowed us to pre-determine the preferred direction of the MF unit under investigation and use that direction as the rewarded direction in which the centrifugal saccades were made. A majority (115 out of 117) MF units investigated in this study exhibited a much stronger (“burst-type”) discharge during the peri-saccadic period in their preferred direction (=centrifugal direction) as compared to the opposite, non-preferred direction (=centripetal direction) in which very few or almost no spikes fired, resulting in weak modulations. Therefore, MF responses only in the centrifugal direction were considered for classification and all analyses. In the other two units, we did not observe a peri-saccadic burst.

Overall, we observed two main types of burst modulations: the eye position-related tonic discharge preceded by a saccade-related burst, i.e., the “burst-tonic” type, and the saccade-related burst discharges that remained mostly silent outside the peri-saccadic period, i.e., “phasic” type. In order to identify the “burst-tonic” responses, we first identified those units in which the difference between the average firing rate in the post-saccadic period (150 to 250 ms from saccade onset) and the pre-saccadic period (−250 to −150 ms from saccade onset) was larger than 1.5 × standard deviation (SD) of the average firing rate during the pre-saccadic period. Next, we compared the slope values of the linear regression fits applied on the pre-and post-saccadic firing responses, and only those cases in which no significant difference between the slopes was observed, were labeled as “burst-tonic” responses (*n* = 24; Fig. 2a, b; see BT). In other words, if the post-saccadic MF activity was not only larger than the pre-saccadic activity but also remained elevated after the saccade-related burst discharge, the unit’s response was classified as a “burst-tonic” type. The “phasic bursts,” on the other hand, were further categorized into “long-lead burst” types and “short-lead burst” types, based on the timing of each MF unit’s burst modulation onset relative to saccade onset^25^. For this, modulation onsets were detected whenever the averaged MF response crossed a threshold (defined as 3 × SD of baseline activity during −400 to −200 ms from saccade onset). To this end, all MF units in which the burst modulation led the saccade onset by more than 15 ms were labeled as “long-lead burst” types (*n* = 60; Fig. 2a, b; see LLB), whereas those that started firing less than 15 ms before the saccade onset were classified as “short-lead burst” (SLB) types (*n* = 27; Fig. 2a, b; see SLB). The value 15 ms was chosen, based on the observed SD value of modulation onsets of “long-lead burst” MF units identified by Ohstuka and Noda^25^. Given that the timing of the detected modulation onsets was a crucial factor in separating these two categories, in addition to the clarity of their firing patterns, spike data were not smoothened using a Gaussian kernel, as in the case of SSs. Based on this criteria, four units (in addition to two non-bursting units) could not be categorized into any of the three categories as in those cases the onset of burst modulation occurred after (i.e., lagged) saccade onset. The timing of the modulation offsets was detected whenever the averaged MF response dropped below the threshold value (defined as 3 × SD of baseline activity during 200–400 ms from saccade end) during the post-saccadic period.

### Classification of simple spike responses

SS responses of individual PCs were broadly categorized into four types—burst, pause, burst-pause and pause-burst—based on their pattern of firing during the perisaccadic period of −50 to 150 ms from the end of primary saccades (note: all primary saccades between 13 and 17 degrees of amplitude were detected using a velocity threshold of 30 deg/s). To this end, we estimated the mean spike density function of the SS discharge of individual PCs by first convolving the time of each SS event detected within a trial with a normalized Gaussian kernel (SD = 5 ms) and then averaging across all trials.

We found that in almost 50% of PCs the SS firing patterns were entirely different for saccades made in the centrifugal and centripetal directions. For instance, a PC could demonstrate a sharp peri-saccadic increase (or burst) in SS firing for a rightward centripetal saccade, whereas in the opposite direction (i.e., left centrifugal) the same PC could exhibit a sudden drop (or pause) in SS firing. Therefore, only for the purpose of demonstrating these different response categories and their corresponding modulations with respect to changes in saccade kinematics (shown in Fig. 3) we considered the response of each PC separately for each tested direction. Consequently, every PC contributed to each response category at least once if the firing patterns were different in the centrifugal and centripetal directions, and twice if they were same (i.e., *n* = 302; 151 PCs × 2 directions). Whereas the quality of our results shown in Fig. 3 may have benefited from such a treatment, the impact of potentially unknown variables cannot be completely ruled out. To avoid this risk, we only consider the SS activity of saccades made in centrifugal (left or right) direction for all analyses, that appear later for the calculation of SS manifolds for the pseudo-population of PCs.

We describe our classification procedure as follows. As the first step, we used a threshold-based criteria to label each SS response with one of the four types based on the polarity of the response modulation. For this, we identified all maximum (peaks) and minimum (troughs) SS firing rates (detected using the MATLAB function “findpeaks,” minimum peak distance = 10 ms, minimum peak prominence = 2 spikes/s) during the peri-saccadic period. The modulation was considered significant if the peaks and troughs crossed an upper and a lower threshold (defined as ±5 × SD of baseline activity during the −250 to −100 ms from saccade onset), respectively. The SS response was classified as a “burst” or a “pause” type if we encountered only a monophasic increase or decrease in SS firing during the peri-saccadic period. Responses were categorized into “burst-pause” or a “pause-burst” types if the first modulation in the biphasic responses showed an increase (followed by decrease) or a decrease (followed by increase) in SS firing, respectively. Next, we ran a principal component analysis (PCA) on the 302 SS responses (centrifugal and centripetal directions combined) to obtain a 2D plot (Fig. 3a) of their first two principal components (explaining 62.2% of the total variance) that seemed to appear as overlapping clusters organized in a circular pattern, centered around the origin. For better discrimination of these clusters, we relied on the SS response labels (identified in the first step) to obtain decision boundaries by resorting to linear discriminant analysis (LDA). As shown in Fig. 3a (dashed lines), the first decision boundary separated the “burst” (blue cluster) from “burst-pause” (green cluster) types, as well as the “pause” (orange cluster) from the “pause-burst” (red cluster) types. On the other hand, the second decision boundary separated the “burst” from “pause-burst” types, and the “pause” from the “burst-pause” types. As compared to the threshold-based labeling of these response patterns, the LDA approach was clearly better in separating these response types (Supplementary Fig. 2c, d).

### Rate models for individual MFs and PCs

We constructed the firing rate model of individual MFs and PCs by using a linear combination of kinematics-independent and kinematics-dependent components. Given the baseline-subtracted dynamic firing rate of the *n*^th^ unit, *R*_n_(*t*,**z**), where *t* is the time from saccade onset and **z** is a vector of the specific movement kinematic parameter (e.g., **z** = [PV] or [duration], or a pair of kinematic parameters, i.e., **z** = [PV, duration]), we modeled the firing rate vector of a “pseudo-population” containing *N* number of neurons, **R**(*t*,**z**) = [*R*_1_(*t*,*z*); *R*_2_(*t*,*z*);…; *R*_*N*_(*t*,*z*)], as

1 $${{{{{\bf{R}}}}}}\left(t,{{{{{\bf{z}}}}}}\right)={{{{{{\bf{R}}}}}}}_{0}\left(t\right)+\mathop{\sum}\limits_{z}\delta z\,{\partial }_{z}{{{{{\bf{R}}}}}}\left(t\right)$$

where **R**_0_ and *∂*_*z*_**R** are the kinematics-independent and kinematics-dependent part, respectively, and *δz*=*z*−*z*_0_ is the deviation of *z* from the mean value of *z, z*_*0*_. We used the multivariate linear regression of the firing rate data with respect to the kinematic parameters (Supplementary Fig. 3a) for each unit to find the model components for all unit data (Supplementary Fig. 3b, c). See Supplementary Methods for details.

### Estimation of manifolds

To find the dimensionally reduced approximation of the population rate model, **R**(*t*,**z**) in Eq. 1, given by **R**_0_ and *∂*_*z*_**R**, we followed the following steps: First, **R**_0_ and *∂*_*z*_**R** were converted to (*N*,*T*) matrices by discretizing time where *T* is the length in time in milliseconds. Second, we performed PCA on **R**_0_, which contains the mean firing rates of individual units at **z** = **z**_0_ at every time point. We obtained a dimensionally reduced representation, a manifold, **P**_*K*_ such that **R**_0_ ≈ **WP**_*K*_ where **W** is some (*N*,*K*) dimensional matrix (*K* < *N*). We determined *K* by finding the number of dimensions (principal components) capturing >85% of the total variability and confirmed it by the cross-validation analysis. Finally, we estimated the linear approximation of how the kinematics-dependent component, ∂_*z*_**R**, would change the PCA result of the firing rates if **z** deviates from **z**_0_. Our analytic estimation showed that it is enough to consider a change in **P**_*K*_ as,

2 $${{{{{{\bf{P}}}}}}}_{K}\to {{{{{{\bf{P}}}}}}}_{K}+\mathop{\sum}\limits_{z}\delta z\,{\partial }_{z}{{{{{{\bf{P}}}}}}}_{K},\,{{\partial}_{z}{{{{{\bf{P}}}}}}}_{K}={{{{{{\bf{W}}}}}}}^{{{\dagger}} }\left({\partial}_{z}{{{{{\bf{R}}}}}}\right)+{\left({\partial}_{z}{{{\bf{W}}}}\right)}^{{\dagger}} \left({{{{{{\bf{R}}}}}}}_{0}-{{{{{\bf{W}}}}}}{{{{{{\bf{P}}}}}}}_{K}\right)$$

to predict the PCA results of **R**(*t*,**z**) with sufficient accuracy. *∂*_*z*_**W** is a matrix obtained in the second step and describes how the higher dimensional (>*K*) components move into the *K*-dimensional subspace when kinematic parameters change and † represents conjugate transpose. See Supplementary Methods for details.

### Analysis of manifolds

Given a manifold of MFs or PCs for a given kinematic variable, we computed the manifold size and rotation speed in 2D (Figs. 4–5). We defined the manifold size as the area enclosed within the 2D circular trajectory, computed by numerically integrating the areas of triangles defined by two neighboring data points and the origin (0,0). For the rotation speed, we first computed the phase of rotation *θ* at each data point (*x*,*y*) by *θ* = tan^−1^(∆*y*/∆*x*) where (∆*x*, ∆*y*) = (*x−x*_0_, *y*−*y*_0_) and (*x*_0_, *y*_0_) is a reference point defined by [(maximum of *x* coordinate data)/2, 0]. Then, we estimated the time *T*_*3/4*_ from the trial beginning *t* = −250 ms, where *θ* ≈ −180° (by definition), to the point *θ* = 90° (rotation of 3/4 cycles), finally finding the average rotation speed by 270°/*T*_*3/4*_. We summarized how the manifold size and rotation speed vary with the kinematic parameters by computing the normalized slope angle in the manifold size and rotation speed plane (Figs. 4p, q and 5c). To do so, we first normalized the manifold size and rotation speed data for all cases by the standard deviations of the control case, which was the correlated variation in Fig. 4p, q and post-no-CS case in Fig. 5c. The slope angle was computed in each case in the normalized coordinates. We also performed the comparison/alignment analysis of multiple manifolds using the canonical correlation analysis^21,26^. See Supplementary Methods for details.

### Linear feed-forward network models (LFFN)

The LFFN models had movement kinematics-independent and dependent components for output variables (**Y**, ∂_**z**_**Y**) and input (**X**, ∂_**z**_**X**), such as PC and MF firing rates in Fig. 6a, b. We assumed that the movement variable ***z*** follows the Gaussian distribution and estimated the weight matrix **T** to minimize the least-square error,

3 $$E({{{{{\bf{T}}}}}})=||{{{{{\bf{Y}}}}}}-{{{{{\bf{T}}}}}}{{{{{{\bf{X}}}}}}}||^{2}+\mathop{\sum}\limits_{z}\mathop{\sum}\limits_{z'}{{{{{\rm{Cov}}}}}}[z,z']({\partial }_{z}{{{{{\bf{Y}}}}}}-{{{{{\bf{T}}}}}}{\partial }_{z}{{{{{\bf{X}}}}}}).\,({\partial }_{z'}{{{{{\bf{Y}}}}}}-{{{{{\bf{T}}}}}}{\partial }_{z'}{{{{{\bf{X}}}}}}),$$

Performances of all the LFFN models were measured by this least-square error. To prevent overfitting we used the LASSO regression^61^ where the hyperparameter was chosen by AIC minimization. For the manifold-transformation in LFFN (Fig. 6e), we reused **T** from the MF-to-PC LFFN model but replaced the input variables by those approximated by the *d*_MF_-dimensional MF manifold. The communication subspace model (Supplementary Fig. 8d, e) was obtained by the rank-reduced regression^34^ with the error function in Eq. 3. See Supplementary Methods for details.

### Statistical analysis

In most data analyses, we evaluated a mean and standard error of mean (SEM) by the jackknife resampling except for two quantities. In testing the prediction of the population-averaged firing rate by models (Supplementary Figs. 3e, g and 4b, d), we separated trials into two equal-sized sets, trained the model by only one of them (train data), and tested it on the other data set (test data). In Fig. 6g, we used the bootstrap procedure that randomly sampled the goodness of fit for individual time points and computed their averages with 500 repetitions to give the bootstrap mean and SEM.

### Reporting summary

Further information on research design is available in the Nature Portfolio Reporting Summary linked to this article.

## Supplementary information

Supplementary Information

Reporting Summary

## Data Availability

All source data files are provided with this paper and can be downloaded from 10.5281/zenodo.7732421. Source data are provided with this paper.

## Source data

Source Data

## Code availability

All underlying codes to reproduce the results and figures of this paper are available at 10.5281/zenodo.7732421.
