# Supplementary material for: Multidimensional cerebellar computations for flexible kinematic control of movements
Source: Nat Commun. 2023 May 3;14:2548. doi: 10.1038/s41467-023-37981-0 (PMC10156706; doi:10.1038/s41467-023-37981-0)
Supplement: Supplementary file 1 — Supplementary Information [file 41467_2023_37981_MOESM1_ESM.pdf]

Supplementary Material:

Multidimensional cerebellar computations for flexible kinematic control of movements

Akshay Markanday*[1], Sungho Hong*[2], Junya Inoue[1], Erik De Schutter[2], Peter Thier[1]

1. Hertie Institute for Clinical Brain Research, University of Tübingen, Tübingen, Germany
2. Computational Neuroscience Unit, Okinawa Institute of Science and Technology, Okinawa, Japan

* These authors contributed equally

Correspondence

Peter Thier
Cognitive Neurology Laboratory,
Hertie Institute for Clinical Brain Research,
Hoppe-Seyler-Str. 3, 72076
Tübingen, Germany.
Email: thier@uni-tuebingen.de

Supplementary figures

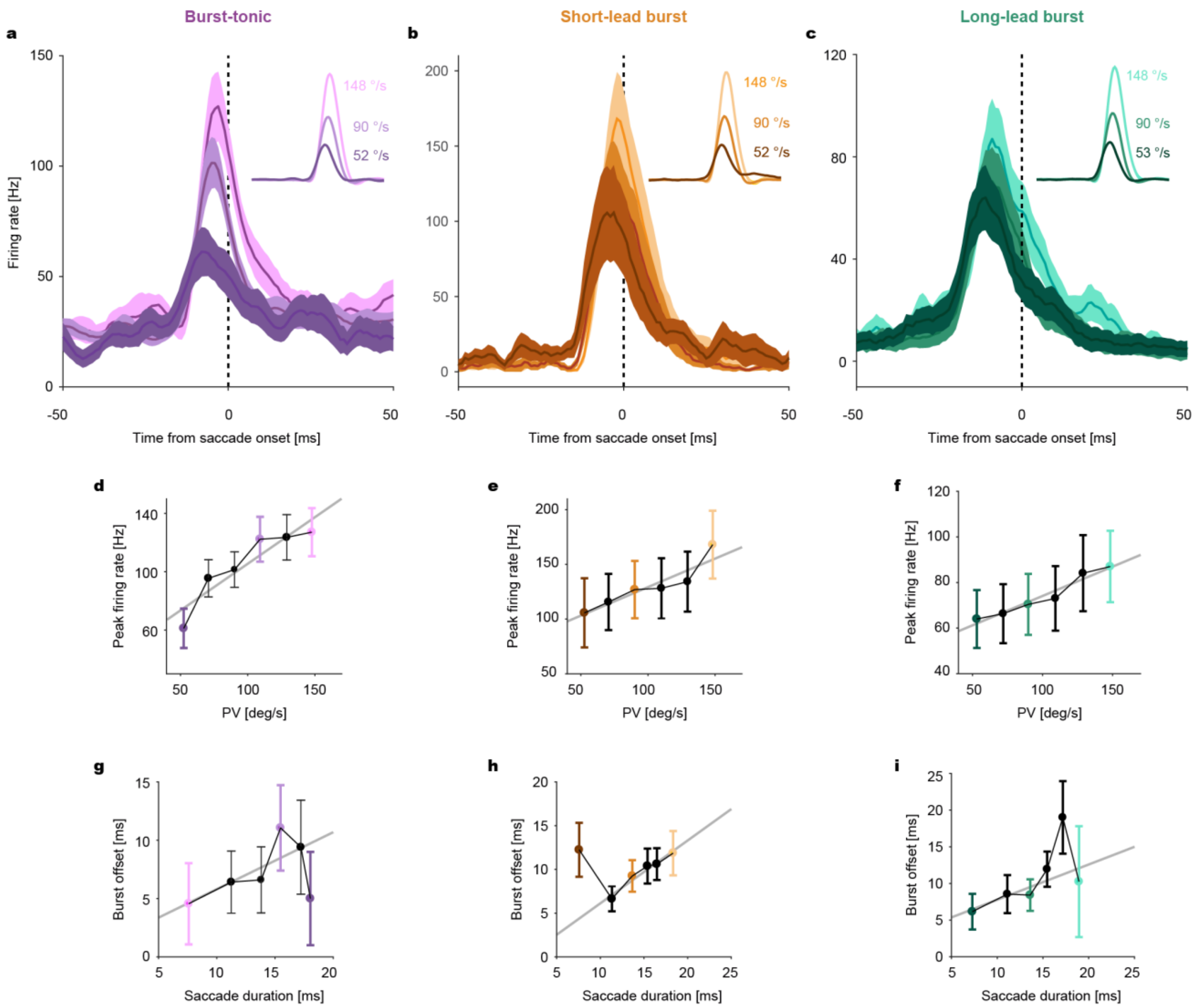

Supplementary figure 1. Linear encoding of the kinematics of microsaccades by mossy fibers (MFs). **a, b, c** Population response of burst-tonic (BT, purple, n=24), short-lead burst (SL, brown, n=27) and long-lead burst (LL, green, n=60) MFs to saccades of different peak velocities (PV, see insets for average velocity profiles), represented by different shades. **d, e, f** Average peak firing rate as a function of saccade peak velocity (bin size=20 deg/s) for each MF category. Linear regression parameters: Burst-tonic: p=0.012, R^2=0.85; Short-lead burst: p=0.01, R^2=0.85; Long-lead burst: p=0.001, R^2=0.95. **g, h, i** Average burst offset relative to saccade onset as a function of saccade duration (calculated from velocity bins) for each MF category. Linear regression parameters, Burst-tonic: p=0.18, R^2=0.21; Short-lead burst: p=0.01, R^2=0.02; Long-lead burst: p=0.22, R^2=0.44. Solid gray lines represent the linear regression fits. Dark and light-colored bins correspond to the high and low peak velocity bins, respectively, for which population responses in a, b and c are plotted for comparison. Data are mean±SEM.

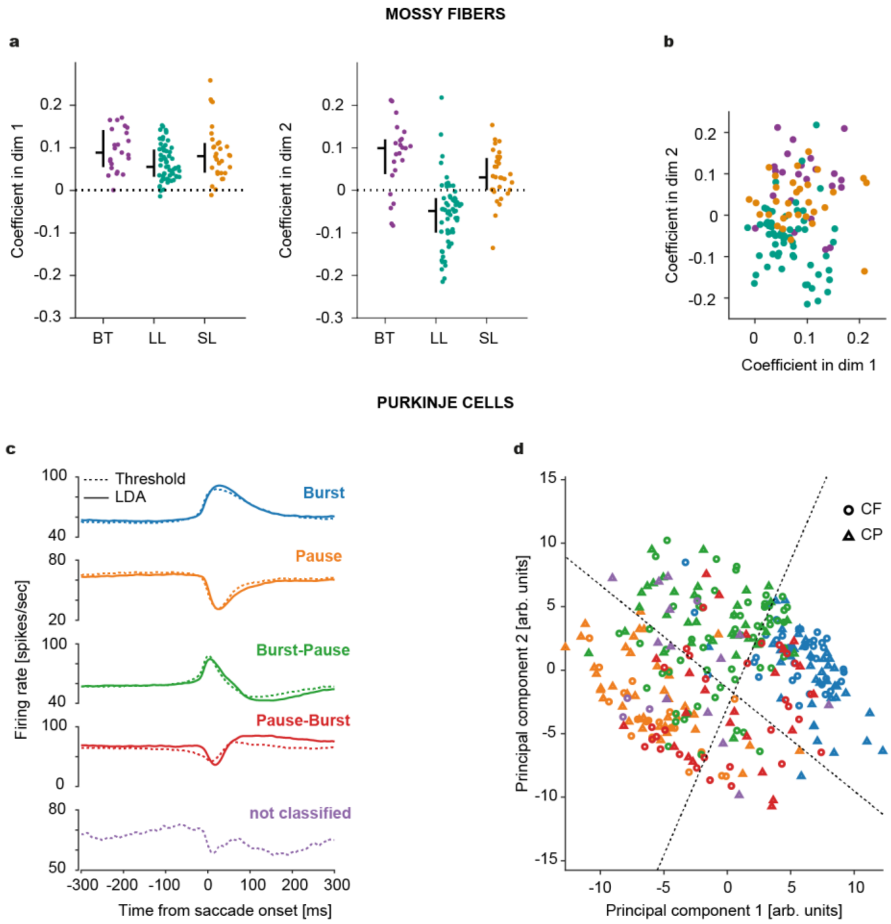

Supplementary figure 2. MF and PC units appear continuous in their distributions. **a** Coefficients of MFs for the first (Left) and second (Right) dimension in the MF manifold. Horizontal bar: median. Vertical bar: Range from the first to third quantile. **b** 2D scatterplot for the coefficients in a. Note a nearly continuous distribution of data points with significant overlaps between BT (n=24 units), SL (n=27 units) and LL (n=60) MF types (denoted by colors). **c** Average firing response of all PCs categorized into burst (n=107 units; blue), pause (n=99; orange), burst-pause (green; 72) and pause-burst (n=24; red) types by threshold-based labeling (dashed lines) and linear discriminant analysis (LDA). Purple dashed lines indicate the average response of those PCs units (n=17) which could not be classified into any of the four categories by the threshold-based method. **d** 2D scatterplot of the coefficients of first two principal components identified by the PCA for individual PC units recorded for centrifugal (CF, circles) and centripetal (CP, triangles) saccades. Dashed lines indicate the decision boundaries estimated by the LDA. Colors represent the PC category. Note, the overlap between different categories.

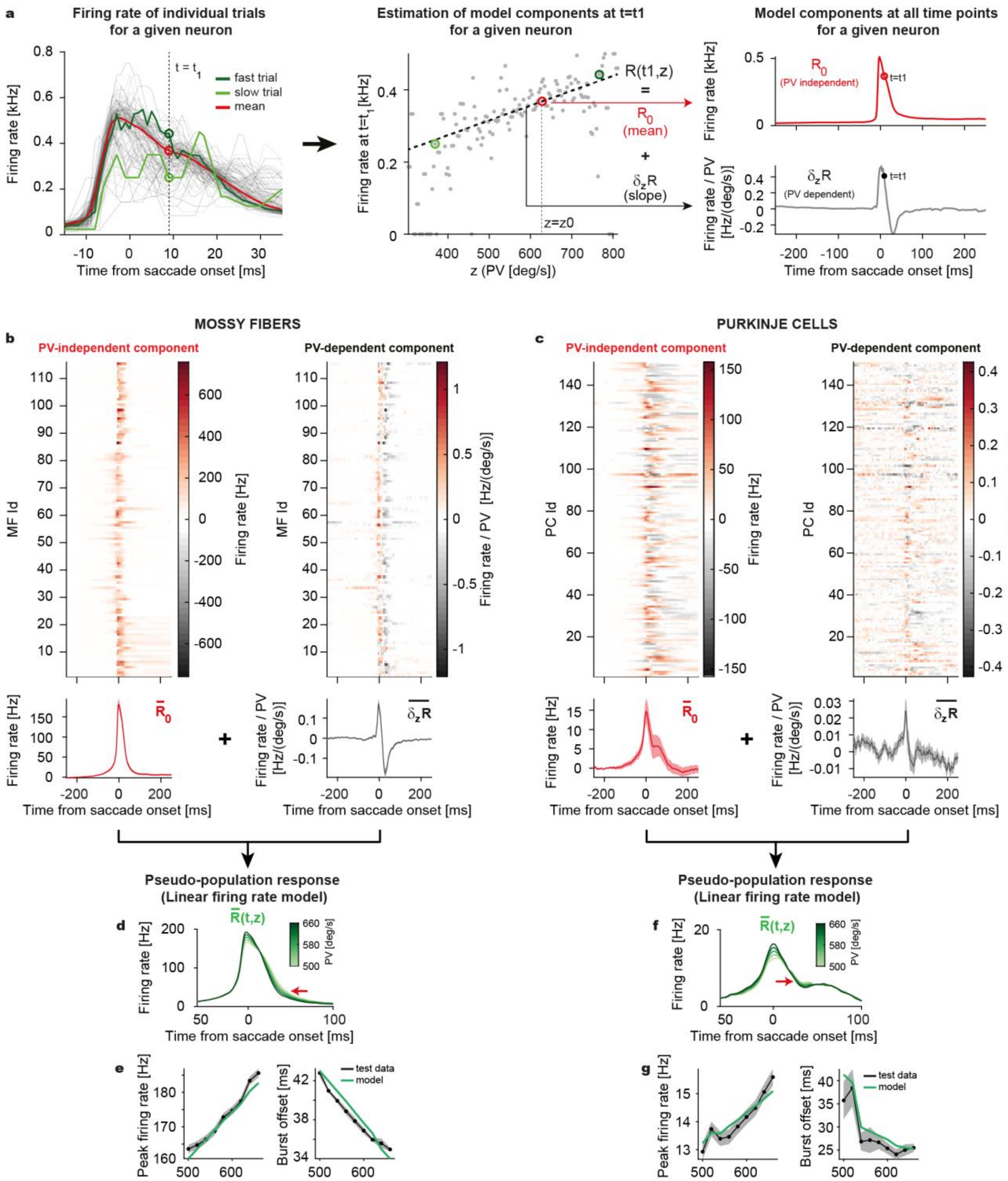

Supplementary figure 3. MF and PC-SS linear firing rate models. **a** Schematic illustration showing the steps involved in the construction of a rate model for individual (MF and PC) units/neurons using PV as the control kinematic parameter for the model. From the time-dependent firing rate estimations for individual trials of a given unit (Left), we create the linear regression model of movement kinematics, such as PV, versus firing rates at each time point (Middle). For example, given a linear dependence of MF or PC-SS firing rates on saccade PV, a randomly chosen saccade with high PV will be associated with higher firing rates (fast trial, dark green) as compared to a low PV saccade (slow trial, light green) and the difference between firing rates will be more pronounced during the initial phase of a saccade. Also, the slopes of regression will be much steeper at time points that fall within the peri-saccadic period

In pre- and post-saccadic periods, where fast and slow trials can no longer be differentiated by PV, the differences in firing rates will also eventually disappear and the slopes will also be flatter. From the center (mean) and slope of the result, we obtain the kinematics-independent and dependent components (Right). **b,c** Top: Heat-map showing PV-independent ($\mathbf{R}_0$) and dependent components ($\partial_{PV}\mathbf{R}$) for individual MF (b) and PC models (c). Bottom: Population averages. The baseline firing rates are subtracted. **d** Pseudo-population average firing rate for different PVs, computed from MF models in **b**. The red arrow indicates the point of burst offset. **e** Average peak firing rate (Left) and burst offset time (Right) vs PV from the models and test data. Goodness of fit: R^2 = 0.929±0.005 (Left), 0.887±0.026 (Right). **f,g** Same plots as d,e but for PCs. R^2 = 0.809±0.023 (Left), 0.619±0.095 (Right). Note that using the PV-and-duration model did not significantly improve the predictions in e,g: peak firing rate vs PV, MFs: R^2=0.929 ± 0.005, PCs: R^2=0.791 ± 0.017; burst offset vs PV, MFs: R^2=0.892 ± 0.021; PCs: R^2=0.702 ± 0.05. Data are mean±SEM.

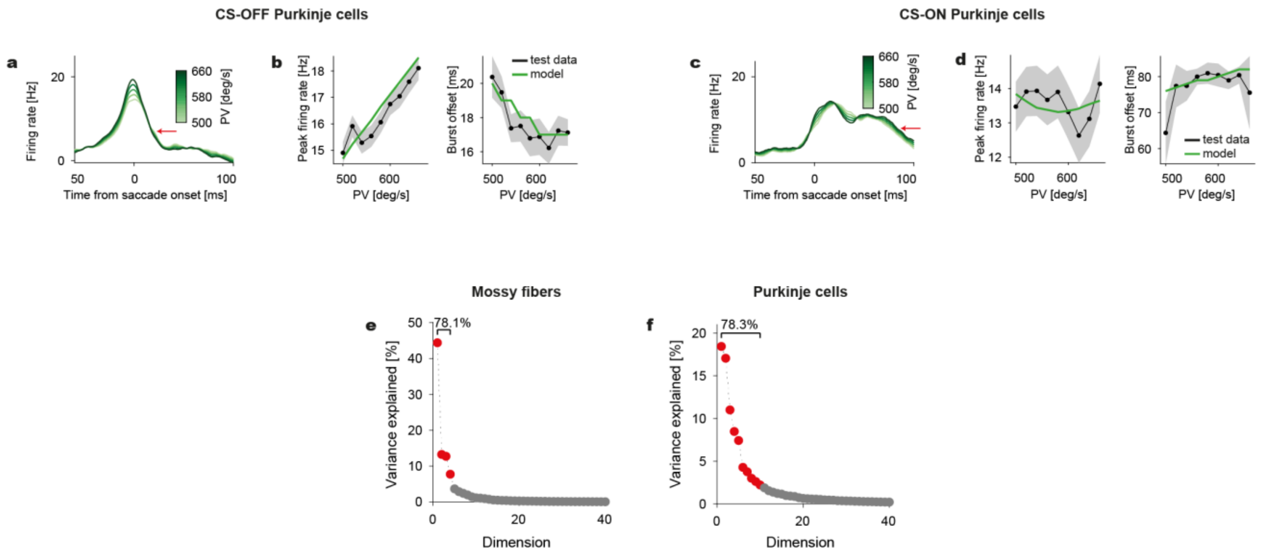

Supplementary figure 4. Pseudo-population SS response for CS-ON and CS-OFF population of PCs. **a** PV-dependent population average firing rates. The red arrow indicates the point of burst offset. **b** Average peak firing rate (Left) and burst offset time (Right) versus PV from the models and test data in CS-OFF direction. Goodness of fit: R^2 =0.689±0.051 (Left), 0.433±0.121 (Right). **c** Same as a, but for CS-ON PCs. **d** Same plots as b but for CS-ON PCs. R^2 = 0.018±0.033 (Left), 0.092±0.088 (Right). The baseline rates are subtracted in all data. **e,f** Variance explained by each dimension in the PCA analysis of the PV-dependent components of the MF (e) and PC-SS models (f). Components with >78% are marked in red. Data are mean±SEM. Note that we did not apply physiologically unjustifiable normalizations to the PV-dependent components for MFs and PCs (inputs to the PCA) since individual units exhibit different discharge rates.

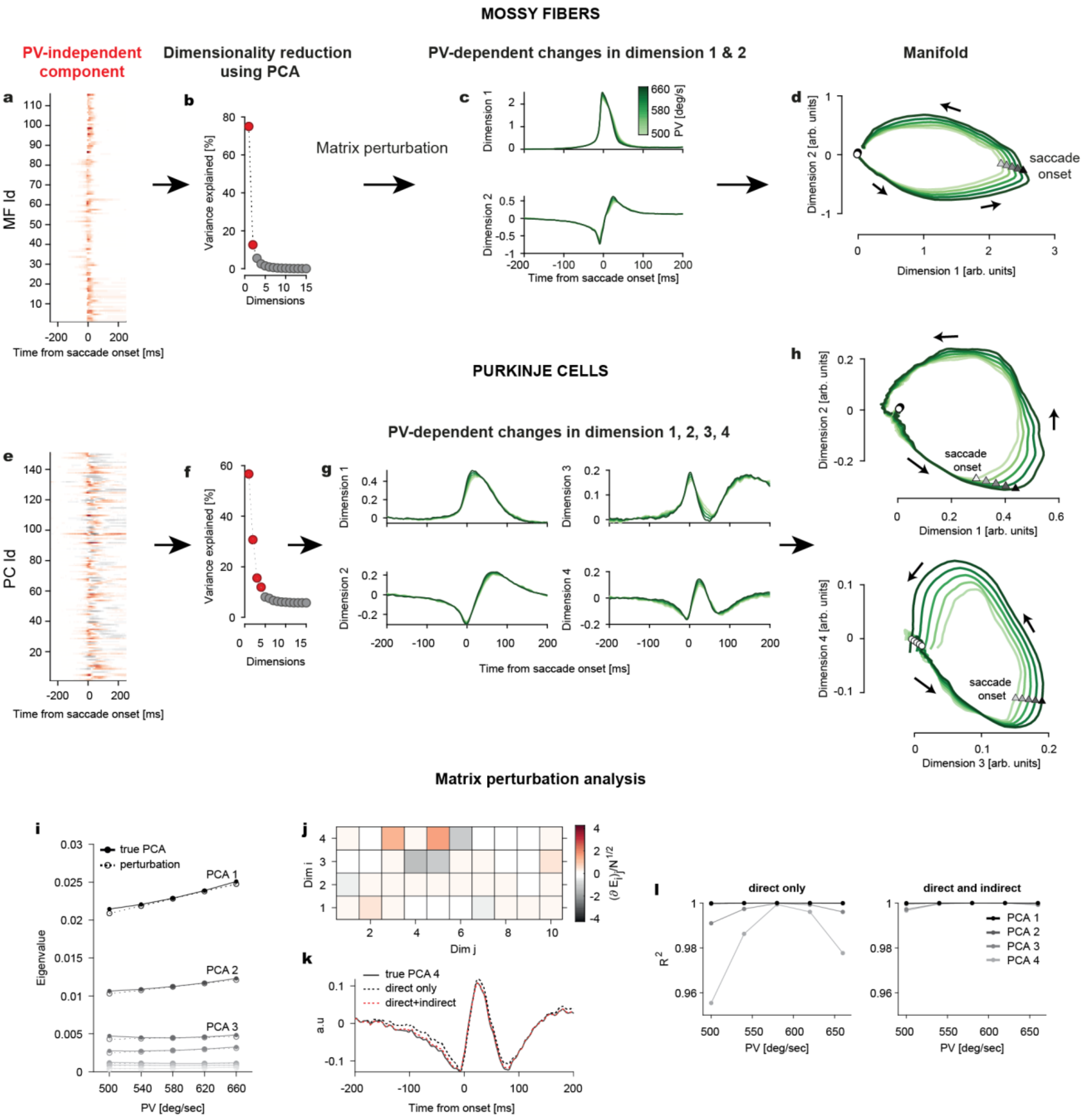

Supplementary figure 5. Step-by-step procedure for identifying manifolds. **a** PV-independent components of all MF units (parameter: PV). First, PCA is performed on the average PV-independent components of individual MF units. **b** The first two principal components (or dimensions, red dots) of the PV-independent components explain a dominant fraction of cell-to-cell variability. **c** Matrix perturbation analysis (see **Methods** and **Supplementary Methods**) computes PV-dependent changes in the first two dimensions, plotted against time. **d** MF manifolds are identified by plotting the first two dimensions against each other for different values of PV (shades of green). Note the separation of curves, depicting increase in manifold size, with increments in PV, both before and after saccade onset (triangles). Arrows indicate the direction of rotation. **e-h** Same as a-d, for PCs. Here, four dimensions explain majority of cell-to-cell variability. Note how the differences in manifold, in the first two dimensions, are limited to periods after saccade onset, whereas in the third and the fourth dimension, changes also appear before saccade onset. The trajectories for the third and fourth dimensions (h, bottom) are plotted only until 50 ms after saccade onset to highlight the changes occurring before saccade onset. **i-l** Approximation of the movement parameter-dependent manifolds,

based on the matrix perturbation theory (**Equation 2** and **Supplementary Methods**), provides good accuracy. For different PVs, the true eigenvalues from PCA of the PC-SS data (solid) match the predictions of the matrix perturbation theory (dotted) well (**i**). The PV-dependent perturbation of the PCA eigenvectors (row) get significant contributions from a limited number of dimensions in the unperturbed eigenvectors (column). The coefficients (color) are normalized by $N^{1/2}$, where N =151 is the number of PCs (**j**). Therefore, even when PV significantly deviates from the mean value (here PV=500°/s in **k**), Equation 2 (red dots) provides a good approximation of the principal components (here the fourth dimension; black solid) while ignoring the eigenvector perturbation (j) (black dotted; see **Supplementary Methods**) will lead to inaccuracy (**k**). This holds for all the principal components of the PC SS data and a wide range of PVs (**l**).

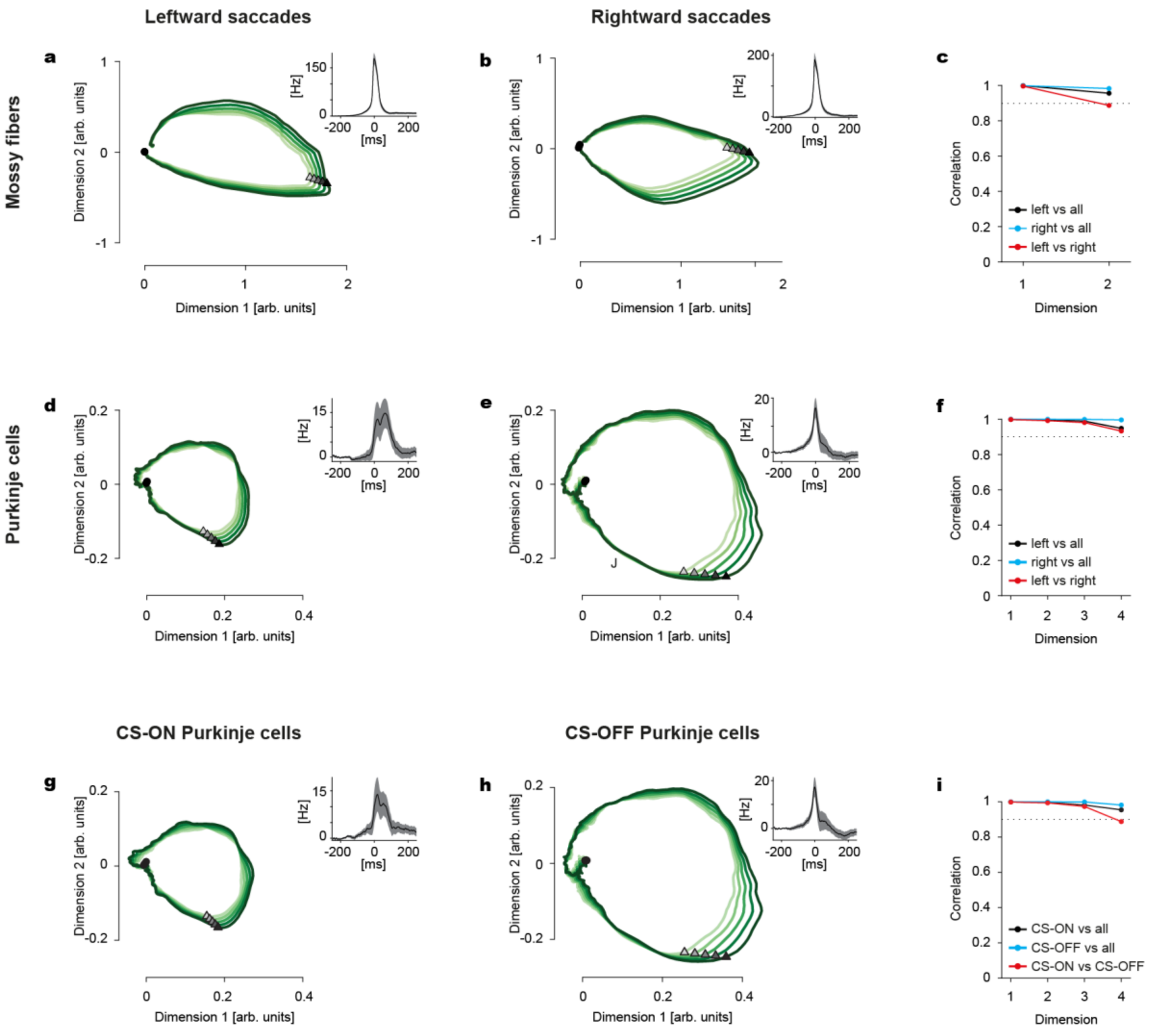

Supplementary figure 6. MF and PC-SS firing rate models and manifolds for different eye movement directions. **a,b** 2D plots of the MF manifolds from the left- and right-directed saccades. Insets show the population average of the PV-independent components of all MF firing rate models (control parameter: PV). **c** Canonical correlation of each dimension in the MF manifold between the left and right directions. Dotted line represents correlation=0.9. **d-f** Same plots as a-c for PC-SS manifolds. **g-h** 2D manifolds of PC-SSs separately for a population of CS-ON and CS-OFF PCs. Note that the similarity in d and e to g and h, respectively, is because most PCs had their CS-ON in leftward direction and CS-OFF in the rightward direction. **i** Same as f, but for PCs.

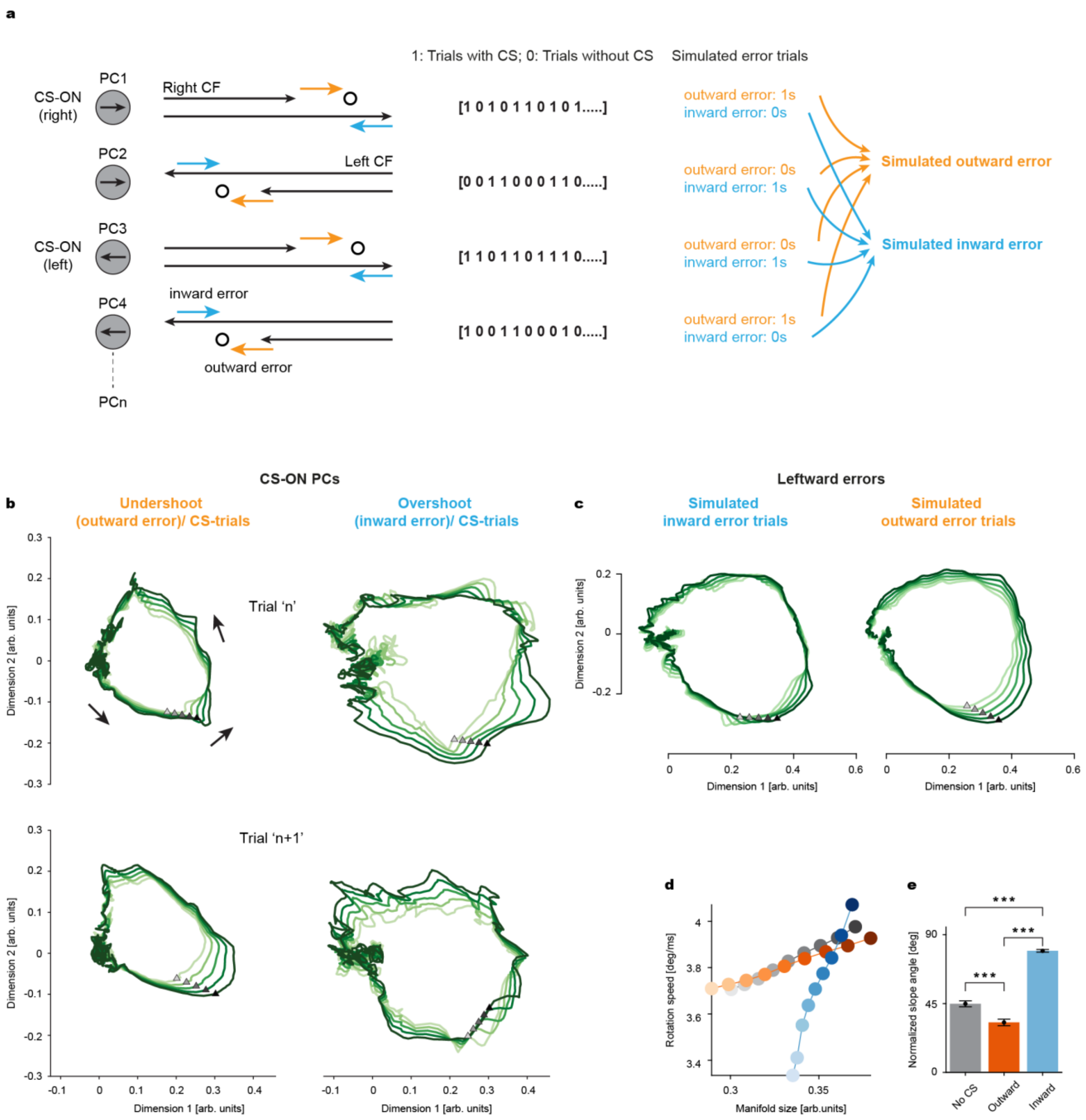

Supplementary figure 7. CSs influence PC-SS manifolds differently, depending on the error type, irrespective of its direction. **a** Illustration showing simulation of inward and outward errors. Gray circles with arrows represent individual PCs with preferred error direction (CS-ON) Black arrows indicate over- and undershooting primary saccades, which could lead to inward (blue arrows) and outward errors (orange arrows) in individual recording sessions. Trials with CS activity (i.e., 'CS-trials') within the post-saccadic period of 50-140 ms are labelled as 1, and 0 otherwise ('no CS-trials'). Depending on a PC's CS-ON direction, every 'CS-trial' is hypothesized to report an inward or outward error, and every 'no CS-trial' should report error in the opposite direction, regardless of whether these errors actually occur or not. We did this for every PC and combined all trials (with and without CSs) that reported outward and inward errors, separately, to determine the influence of CS in the simulated inward and outward error conditions. **b** Up: Manifolds when outward (left) and inward (right) errors occurred in trial 'n' accompanied by CS firing in the post-saccadic period. Down: Manifolds in the subsequent trial 'n+1' change differently for outward and inward errors, like those for simulated error trials shown in Fig. 5b. Filled triangles are saccade onsets and black arrows

indicate the direction of rotation for all manifolds. **c** Manifolds for simulated post-inward and post-outward error trials controlled for error direction (i.e., Leftward errors). **d** Rotation speed as a function of manifold size for simulated post-inward (blue), post-outward (orange) and no-CS control (gray) trials. **e** A comparison of normalized slope angles for each condition. Note that the error-type specific changes in manifolds are preserved, i.e., an outward error-related increase in manifold size (indicated by the relatively flatter slope of the orange curve as compared to No-CS) and inward-error related change in rotation speed (indicated by relatively steeper slope as compared to the No-CS condition), despite the error vector pointing in the same left direction. Data are jackknife mean±SEM from n=151 PCs**.** T-value (No-CS, Outward) =5.93, $p=9.88x10^{-9}$; T-value (Outward, Inward) = -28.28, $p=1.59x10^{-62}$; T-value (No-CS, Inward) =-23.03, $p=1.41x10^{-51}$. P-values are from one-sided Student *t*-tests.

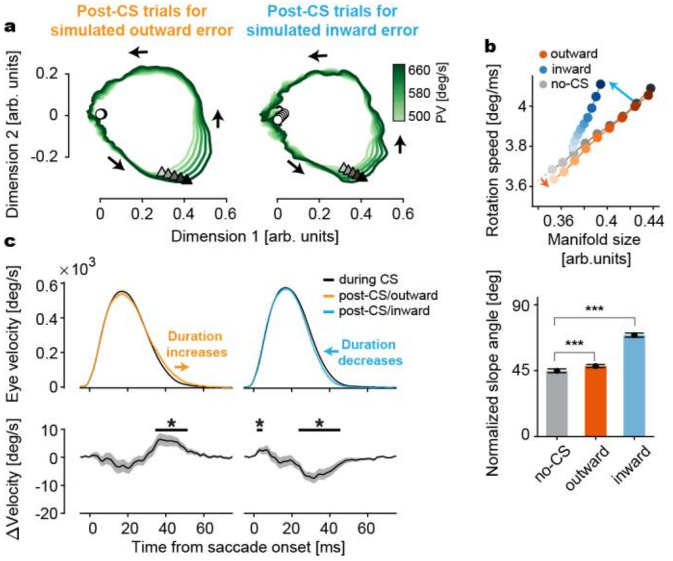

Supplementary figure 8. Influence of CSs during 140-250 ms from saccade offset on subsequent trial's PC-SS manifolds for simulated inward and outward errors. **a** Left: PC manifolds reflecting the combined influence of simulated outward errors on subsequent trials. Right: Same as left, but for inward errors. **b** Top: Manifold size versus rotation speed after the outward (orange) and inward (blue) error-encoding CS-trials, and after no-CS trials (grey). Color bar gradient represents PV from 500 deg/s (brightest) to 660 deg/s (darkest). Bottom: Comparison of normalized slope angles for each condition. Data are jackknife mean±SEM from n=151 PCs**.** T-val (No-CS, Outward) = -2.71; p=3.81x10^{-3}, T-val (Outward, Inward) = -17.18; p=1.22x10^{-37}, T-val (No-CS, Inward) = -17.77; p=4.00x10^{-39}. P-values are from one-sided Student *t*-tests**. c** Top: Average saccade velocity profiles in the CS (black) and post-CS trials (colored) for the simulated outward (Left) and inward (Right) errors. For highlighting the differences in velocity profiles, colored lines represent the cumulative effect of five CSs. Note, how both inward and outward errors are corrected by changing the duration of the subsequent saccade, as suggested by relatively steeper slopes (or larger slope angles) in inward and outward error conditions shown in b. Bottom: Average eye velocity change from the CS to post-CS trials. Data are mean±SEM. *: p<0.05 (two-sided Student *t*-test).

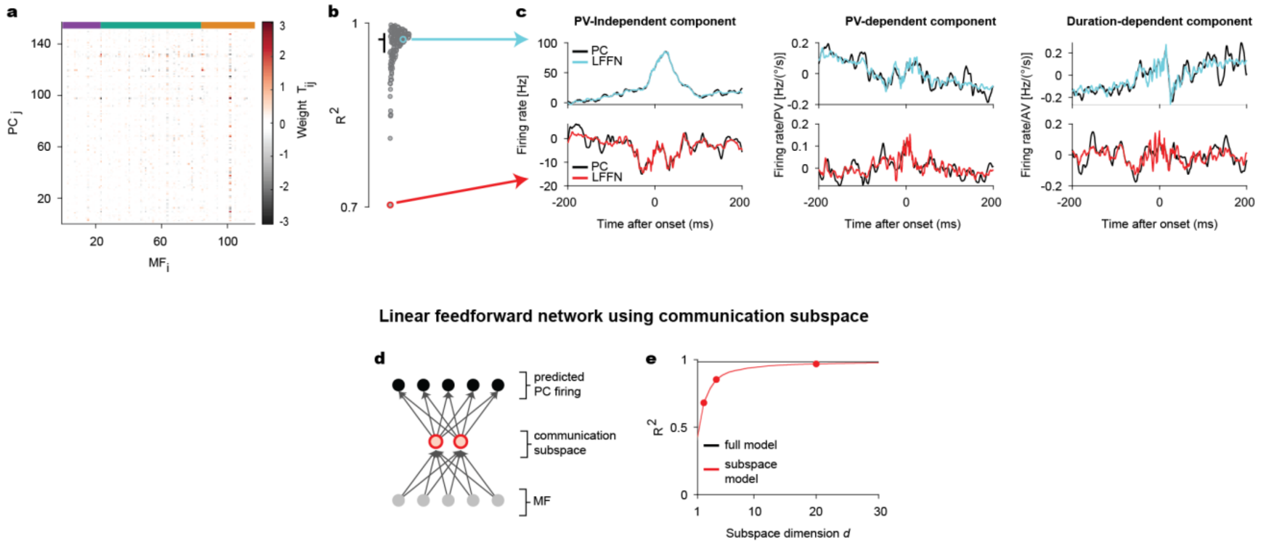

Supplementary figure 9. Linear feed-forward network (LFFN) model for MF-to-PC transformation with PV and duration dependence. **a** Weight matrix of the MF-to-PC network model. **b** Goodness of fit for individual PCs. Colored circles represent the examples in c. Horizontal bar: Median. Vertical bar: Range from the first to third quantile. **c** PV-independent (Left), PV- (Middle), and duration-dependent (Right) component of example PC-SS rate models (black) and prediction by LFFN (color). The baseline rates are subtracted in the PV-independent components. **d** A schematic illustration of the communication subspace model of MF-to-PC transformation. A communication subspace is a (*d*-dimensional) neural subspace of all MF activity that can best predict individual PC-SS rates given dimensionality *d*. See **Supplementary Methods** for how we found the communication subspace given *d*. **e** Goodness of fit for model prediction of PC-SS rates. Red dots represent d=2, 4, and 20. Data are mean±SEM.

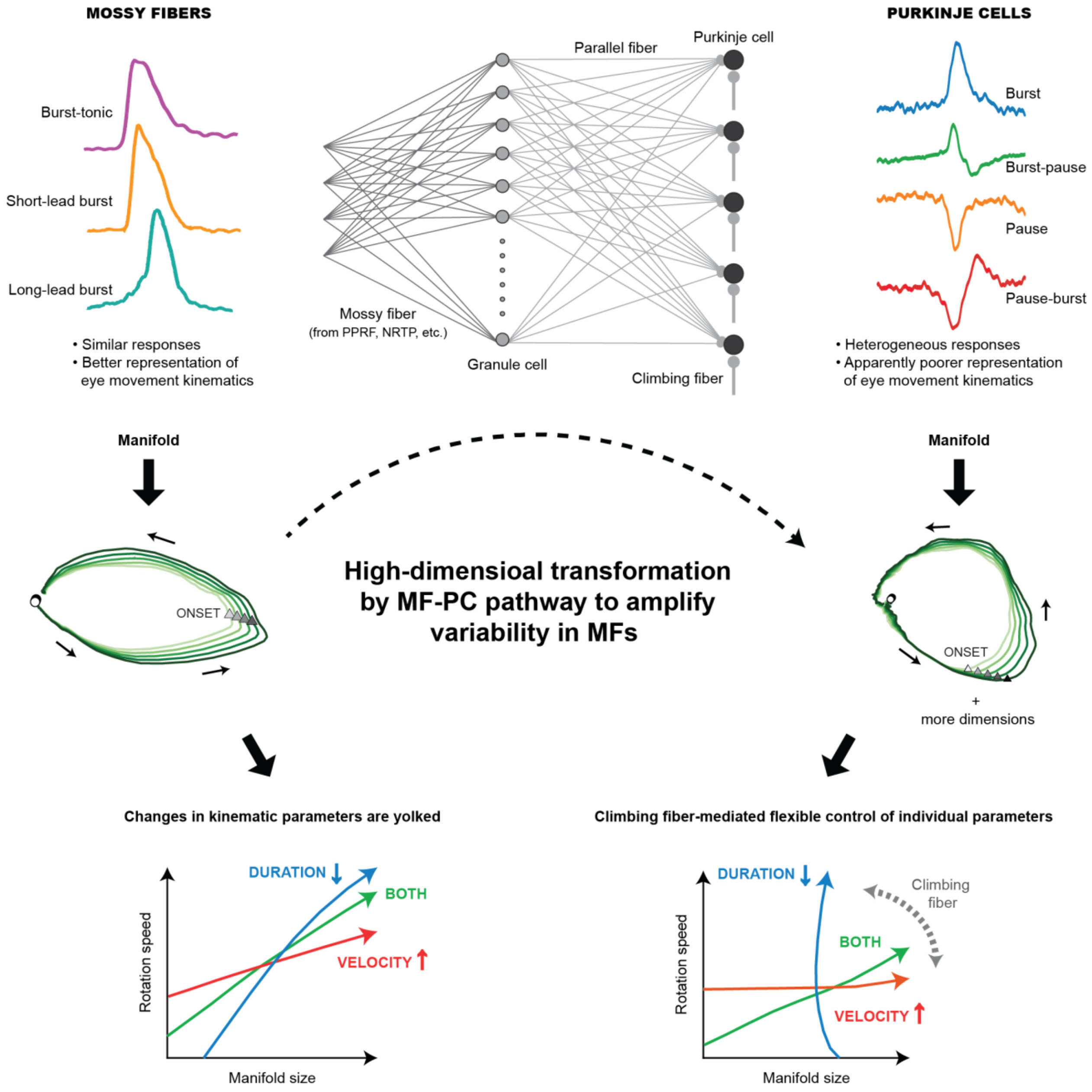

Supplementary figure 10. Summary figure.

Supplementary methods

Fitting the linear rate model to data

In the main text, we modeled the firing rate vector of a "pseudo-population" containing *N* number of neurons, $\mathbf{R}(t,\mathbf{z}) = [R_1(t,\mathbf{z}); R_2(t,v); \ldots; R_N(t,\mathbf{z})]$, as

$$\mathbf{R}(t,\mathbf{z}) = \mathbf{R}_0(t) + \sum_z \delta\, z\, \mathbf{R}_z(t).$$

where $\mathbf{R}_0$ and $\partial_z\mathbf{R}$ are the kinematics-independent and dependent part, respectively. $\delta z = z - z_0$ is the deviation of *z* from the mean value of *z*, z_0.

We fitted this model to the MF and PC data in the following way: We first estimated the firing rate at each trial by the fractional interspike interval method[1] (τ=5 ms). Other estimation methods, such as spike train smoothening by a Gaussian kernel, did not change the results. We sub-selected the firing rate data from *t*=-250 ms to 250 ms and subtracted the baseline firing, estimated by averaging the firing rates from *t*=-250 ms to -150 ms for each trial. By estimating the firing rate for every time bin (=1 ms), our firing rate data became a (N_{trial}, T)-dimensional matrix for each neuron where N_{trial} is a number of trials and T=501, the length of each trial. As for the kinematic parameters, peak velocity (PV) and duration were computed from an eye movement velocity profile for each trial. However, the distribution of saccade duration was skewed and can lead to inaccuracy in regression. In estimation, therefore, we used average velocity (AV), defined by 15°/(saccade duration), as a regression variable, instead of duration since the AV distribution was significantly more symmetric. Finally, we performed the multivariate linear regression of the firing rate data for the kinematic parameters (**Supplementary fig. 3a**) for each unit to find the model components for all unit data (**Supplementary fig. 3b,c**).

We checked the explanation power of the model fitted to each unit data, especially $\partial_z\mathbf{R}$, by computing the Akaike information criterion (AIC). We found that AIC decreased significantly (*P*<0.01, Student t-test) in all units when the PV-dependence is added ($\partial_{\mathrm{PV}}\mathbf{R}$) (MF: ΔAIC=-65.33 $\pm$ 5.39, PC: -23.76 $\pm$ 1.65). Also, AIC significantly decreased (*P*<0.01, Student t-test) in a majority of the units (MF: *n*=99, PC: 83) when duration-dependence is augmented (MF: ΔAIC=-23.60 $\pm$ 3.03, PC: -3.41 $\pm$ 0.48). Therefore, we confirmed that the model captured the true kinematic parameter-dependent trial-to-trial firing rate variability, not the data noise.

We also tested whether the models can predict the average firing rate profile. To do so, we split each data set into two, the training and test data. Then, we first constructed the rate models based on the training data sets. All the trials in the test data are split based on PV bins whose centers were 500°/s, 520°/s, …, 660°/s and widths were 50°/s. We computed the PV-dependent average firing rate time series based on the estimated firing rates from the spike times of all the trials belonging to the PV bins. For each trial, we also computed the rate prediction from the training data and computed the prediction of the average firing rate in the same way as the test data (**Supplementary fig. 3d,f**). To test their agreement, we evaluated the peak firing rate and burst offset time as test measures for the average firing rates from the test data and model prediction (**Supplementary fig. 3e,g**). This procedure was carried out for two different types of models; first, those parametrized only by PV (i.e., **z**=[PV]) and the others with PV and duration (i.e., **z**=[PV, AV=15°/(duration)]). We observed only an insignificant increase in the model performance by including duration as a parameter: With the PV-only model, R^2 for the peak firing rate versus PV were 0.929 $\pm$ 0.005 and 0.892 $\pm$ 0.023 for MFs and PCs, respectively. For the burst offset versus PV, R^2=0.887 $\pm$ 0.026 for MFs and 0.619 $\pm$ 0.095. With the PV-and-duration model, R^2 for the peak firing rate versus PV were 0.929 $\pm$ 0.005 for MFs and 0.791 $\pm$ 0.017 for PCs. For the burst offset versus PV, R^2=0.892 $\pm$ 0.021 for MFs and 0.702 $\pm$ 0.05 for PCs.

Dimensionality Reduction by Principal Component Analysis with Perturbation

We developed a simple variant of principal component analysis (PCA) to perform dimensionality reduction of the population firing rate model. We first assume that, with any specific kinematic parameters (in the range of experimental observations), we can find a good dimensionally reduced representation by performing PCA on the population firing. Then, we find an approximation of the population firing and its change with kinematic parameters by another dynamical process with lower dimensionality. Our goal is that if we perform PCA on this approximated population firing, the result will be sufficiently close to that of the original population firing for any kinematic parameters. In our experimental data, the trial-to-trial variabilities of kinematic parameters and firing rate are relatively small. In this case, we can use the matrix perturbation theory to find such an approximation of the population firing.

Our result can be summarized by Equation 2 in **Methods**: Given the kinematic parameter z, if the time-dependent population firing $\mathbf{R}(t,\mathbf{z})$ of N neurons is described by a linear model in Equation 1, it can be approximated by the K-dimensional vectors $\mathbf{P}_K$ and $\partial_z\mathbf{P}_K$ (K<N) as

$$\begin{aligned} \mathbf{R}(t,\mathbf{z}) &\approx \mathbf{W}(\mathbf{P}_K + \textstyle\sum_z \delta z\ \partial_z\mathbf{P}_K) + \mathbf{R}_\perp, \\ \partial_z\mathbf{P}_K &= \mathbf{W}^\dagger(\partial_z\mathbf{R}) + (\partial_z\mathbf{W})^\dagger(\mathbf{R}_0 - \mathbf{W}\mathbf{P}_K). \end{aligned} \tag{S1}$$

where $\mathbf{W}$ and $\partial_z\mathbf{W}$ are the weight matrices, as long as $\mathbf{R}_0$ admits $\mathbf{R}_0 \approx \mathbf{W}\mathbf{P}_K$ (**Supplementary figure 5a,b** and **e,f**) and $\partial_z\mathbf{R}$ is sufficiently small. Furthermore, when we perform PCA on $\mathbf{R}$, the result is dominated by the first term since $\mathbf{R}_\perp$ makes a negligible contribution (see below). Therefore, we performed most of our manifold analysis in terms of $\mathbf{P}_K + \sum_z \delta z\ \partial_z\mathbf{P}_K$ (**Supplementary figure 5c,d** and **g,h**), without considering $\mathbf{R}_\perp$. An exception is the dimensionally reduced MF firings given to the linear feed-forward network as inputs (**Figure 6**). Here we use the full Eq. S1 for the approximate firing rates of individual neurons.

Estimation of the model components

In the first step, we performed PCA on the kinematic-independent component, $\mathbf{R}_0$ (**Supplementary figure 5a,b** and **e,f**). To do so, we computed the covariance matrix, $\mathbf{C} = \mathrm{Cov}[\mathbf{R}_0(t)]_t$ and its eigenvalues $\{\lambda_n\}$ ($\lambda_i \geq \lambda_j$ for $i > j$) with the corresponding eigenvectors $\mathbf{E} = [\mathbf{E}_1, \mathbf{E}_2, \dots, \mathbf{E}_N]$. If the first *K<N* eigenvalues are dominant (see below for the determination of *K*), a dimensionally reduced approximation of $\mathbf{R}_0$ can be obtained by the projection of the population activity to a *K*-dimensional subspace of $\mathbf{E}$ as

$$\mathbf{R}_0 \approx \mathbf{W}\mathbf{P}_K, \quad \mathbf{P}_K = \mathbf{W}^\dagger\mathbf{R}_0, \quad \mathbf{W} = [\mathbf{E}_1, \dots, \mathbf{E}_K].$$

Then, in the second step, we approximated the full covariance matrix of $\mathbf{R}(t,\mathbf{z})$ as

$$\hat{\mathbf{C}} = \mathrm{Cov}[\mathbf{R}(t,\mathbf{z})]_t \approx \mathbf{C} + \sum_z \delta z\ \partial_z\mathbf{C}, \quad \partial_z\mathbf{C} = \mathrm{Cov}[\mathbf{R}_0, \partial_z\mathbf{R}]_t + \mathrm{Cov}[\partial_z\mathbf{R}, \mathbf{R}_0]_t,$$

assuming that the kinematics-dependent part is sufficiently small. When the second δz-dependent terms are small, they can be considered as small perturbations. In that case, the Rayleigh–Schrödinger perturbation theory tells that eigenvalues $\{\hat{\lambda}_n\}$ and eigenvectors $\hat{\mathbf{E}} = [\hat{\mathbf{E}}_1, \dots, \hat{\mathbf{E}}_N]$ of $\hat{\mathbf{C}}$ are approximately[2,3],

$$\begin{aligned} \hat{\lambda}_n &\approx \lambda_n + \textstyle\sum_z \delta z\ \partial_z\lambda_n, \qquad & \partial_z\lambda_n &= \mathbf{E}_n^\dagger(\partial_z\mathbf{C})\mathbf{E}_n, \\ \hat{\mathbf{E}}_n &\approx \mathbf{E}_n + \textstyle\sum_z \delta z\ \partial_z\mathbf{E}_n, \qquad & \partial_z\mathbf{E}_n &= \textstyle\sum_{k\neq n} \frac{\partial_z\lambda_n}{\lambda_n - \lambda_k}\mathbf{E}_k. \end{aligned} \tag{S2}$$

Supplementary figure 5i shows an example of the PC data. The change in the eigenvalues of the covariance matrix is well approximated by Eq. S2. **Supplementary figure 5j** shows how much contribution each $\partial_z\mathbf{E}_i$ gets from $\mathbf{E}_j$. We can see that $\partial_z\mathbf{E}_3$ and $\partial_z\mathbf{E}_4$ especially get significant contributions not only from the first four $\mathbf{E}_j$'s but also from the higher (*K*>4) dimensional components.

Finally, by using the eigenvector perturbation in Eq. S2, we determined the rest of the components in Eq. S1,

$$\partial_z \mathbf{W} = [\partial_z \mathbf{E}_1, \partial_z \mathbf{E}_2, \dots, \partial_z \mathbf{E}_K],$$
$$\mathbf{R}_\perp = -\textstyle\sum_z \delta z\, \mathbf{E}_\perp (\partial_z \mathbf{E}_\perp)^\dagger \mathbf{W}\mathbf{P}_K, \tag{S3}$$

where $\mathbf{E}_\perp = [\mathbf{E}_{K+1}, \dots, \mathbf{E}_N]$, and $\partial_z \mathbf{E}_\perp = [\partial_z \mathbf{E}_{K+1}, \partial_z \mathbf{E}_{K+2}, \dots, \partial_z \mathbf{E}_N]$. We give the detailed derivation in the next section. Therefore, $\partial_z \mathbf{W}$ represents a contribution from the eigenvector perturbation, and we will call it an *indirect projection*, while we will call $\mathbf{W}^\dagger(\partial_z \mathbf{R})$ a *direct projection* term. On the other hand, $\mathbf{R}_\perp$ represents the *K*-dimensional approximation of $\mathbf{R}_0$ rotating out of the *K*-subspace by perturbation and makes a negligible contribution when we perform PCA. **Supplementary figure 5k,l** show that we can well predict the PCA results of the population firing given the changes in a kinematic parameter, PV, without $\mathbf{R}_\perp$, while the indirect projection part can make a substantial contribution.

Determination of $\boldsymbol{K}$

As a final note, we explain how we determined K in the first step: we first found K components that explained more than 85% of the total variance in $\mathbf{R}_0$. This criterion gave us K = 2 and 4 for MFs and PCs, respectively. We also computed the participation ratio[4,5], $(\sum_n \lambda_n)^2 / \sum_n \lambda_n^2$, which estimated K=2 (MFs) and 3 (PCs), from $\mathbf{R}_0$. However, K=4 for PCs was more robust when we varied kinematic parameters or hyperparameters such as the smoothing time scale for rate estimation. Finally, the cross-validation analysis for PCA[6] of $\mathbf{R}_0$ also confirmed *K*=2 and 4: We first randomly selected 70% of elements in $\mathbf{R}_0$ matrix ("test data") and replaced them by Gaussian random numbers, leaving the other 30% of the "training data" untouched. Using the data with random replacements, we repeatedly performed PCA until we got the stable prediction of the test data. Then, we computed the cross-validation error by the squared sum of the differences between the predicted and real test data. This procedure was repeated 200 times for each *K* from 2 to 20. We found that the cross-validation error, averaged over the repetitions, was minimal at *K*=2 and 4 for the MF and PC data, respectively.

Derivation of Eq. S1 and S3

Given the perturbed eigenvectors in Eq. S2, the projection of the population activity to them, $\widehat{\mathbf{P}}$, is

$$\widehat{\mathbf{P}} = \widehat{\mathbf{E}}^\dagger \mathbf{R} \approx \mathbf{P} + \sum_z \delta z \{\mathbf{E}^\dagger(\partial_z \mathbf{R}) + (\partial_z \mathbf{E})^\dagger \mathbf{R}_0\}$$

where $\mathbf{E} = [\mathbf{E}_1, \dots, \mathbf{E}_N]$ and $\partial_z \mathbf{E} = [\partial_z \mathbf{E}_1, \dots, \partial_z \mathbf{E}_N]$. If the left inverse of $\widehat{\mathbf{E}}^\dagger$ is $\widehat{\mathbf{U}} = \mathbf{U} + \sum_z \delta z\ \partial_z \mathbf{U} + O(\delta z^2)$, we get $\mathbf{U} = \mathbf{E}$ and $\partial_z \mathbf{U} = -\mathbf{E}(\partial_z \mathbf{E})^\dagger \mathbf{E}$, from the condition $\widehat{\mathbf{U}}\widehat{\mathbf{E}}^\dagger = \mathbf{1}$.

Now we find the low dimensional representation $\widehat{\mathbf{P}}_K$ by keeping only the first K components of $\widehat{\mathbf{P}}$, i.e. $\widehat{\mathbf{P}}_K = (\widehat{\mathbf{P}})_K$ where $(\cdot)_K$ denotes selecting only the first K rows in a matrix. With $\widehat{\mathbf{W}} = [\widehat{\mathbf{U}}_1, \widehat{\mathbf{U}}_2, \dots, \widehat{\mathbf{U}}_K]$ and again $\mathbf{W} = [\mathbf{E}_1, \mathbf{E}_2, \dots, \mathbf{E}_K]$,

$$\mathbf{R} \approx \widehat{\mathbf{W}}\widehat{\mathbf{P}}_K = \mathbf{W}\mathbf{P}_K + \sum_z \delta z \{\mathbf{W}(\partial_z \mathbf{P})_K + (\partial_z \mathbf{U}_\parallel)\mathbf{P}_K\} + O(\delta z^2)$$

where $\partial_z \mathbf{U}_\parallel = [\partial_z \mathbf{U}_1, \partial_z \mathbf{U}_1, \dots, \partial_z \mathbf{U}_K]$.

Through a little algebra, this equation can be rewritten as

$$\mathbf{R} \approx \mathbf{W}\mathbf{P}_K + \sum_z \delta z\, \mathbf{W}\mathbf{W}^\dagger(\partial_z \mathbf{R}) + \sum_z \delta z\, \mathbf{W}(\partial_z \mathbf{W})^\dagger(\mathbf{R}_0 - \mathbf{W}\mathbf{P}_K) - \sum_z \delta z\, \mathbf{E}_\perp(\partial_z \mathbf{E}_\perp)^\dagger \mathbf{W}\mathbf{P}_K$$

where $\partial_z \mathbf{W} = [\partial_z \mathbf{E}_1, \partial_z \mathbf{E}_2, \dots, \partial_z \mathbf{E}_K]$. $\mathbf{E}_\perp$ and $\partial_z \mathbf{E}_\perp$ are both orthogonal to the K-subspace as $\mathbf{E}_\perp = [\mathbf{E}_{K+1}, \dots, \mathbf{E}_N]$, and $\partial_z \mathbf{E}_\perp = [\partial_z \mathbf{E}_{K+1}, \partial_z \mathbf{E}_{K+2}, \dots, \partial_z \mathbf{E}_N]$. Defining $\mathbf{R}_\perp = -\sum_z \delta\, z\, \mathbf{E}_\perp (\partial_z \mathbf{E}_\perp)^\dagger \mathbf{W} \mathbf{P}_K$, we reach Eq. S1 and S3.

Alignment of Two Manifolds by Canonical Correlation Analysis

In the main text, we compared manifolds obtained from two or more different data sets, such as firings recorded with the left-directed saccades and those with the right-directed ones. We used canonical correlation analysis (CCA) to verify if a pair of manifolds can be related by a linear transformation[7-9]. We first used the CCA for the kinematics-independent components of two data sets to find the best linear alignment transformation between them. Then, we transformed the kinematics-dependent parts by the alignment transform and checked how well they match with each other.

If there are two data called A and B, we denote the kinematics-independent components ($\mathbf{P}_K$ in Eq. S1) of their manifolds, $\mathbf{P}_A$ and $\mathbf{P}_B$, respectively. Also, we discretize time and consider them as $N \times T$ matrices instead of time-dependent vectors, where T is the length of trials.

First, we perform the QR decomposition,

$$\mathbf{P}_X^\dagger = \mathbf{Q}_X \mathbf{V}_X, \quad X = A, B.$$

From the singular value decomposition of the comparison matrix $\mathbf{Q}_A^\dagger \mathbf{Q}_B = \mathbf{U}_A \mathbf{S} \mathbf{U}_B^\dagger$, we obtain the transformation matrices to the best aligned manifolds, $\widetilde{\mathbf{P}}_A$ and $\widetilde{\mathbf{P}}_B$, as

$$\widetilde{\mathbf{P}}_X^\dagger = \mathbf{P}_X^\dagger \mathbf{M}_X, \quad \mathbf{M}_A = \mathbf{V}_X^{-1} \mathbf{U}_X, \quad X = A, B.$$

The diagonal elements of $\mathbf{S}$ are correlations between $\widetilde{\mathbf{P}}_A$ and $\widetilde{\mathbf{P}}_B$. We obtain a representation of $\mathbf{P}_B$ aligned to A

$$\mathbf{P}_{B\to A}^\dagger = \mathbf{P}_B^\dagger \mathbf{T}_{B\to A}, \quad \mathbf{T}_{B\to A} = \mathbf{M}_B \mathbf{M}_A^{-1}/N,$$

where N is a norm of $\mathbf{M}_B \mathbf{M}_A^{-1}$ to maintain the size difference between two manifolds. Then, Eq. S1 becomes

$$\begin{aligned} \widehat{\mathbf{R}}_B &\approx \mathbf{W}_B \left(\mathbf{P}_B + \sum_z \delta\, z\, \partial_z \mathbf{P}_B \right) + \mathbf{R}_\perp \\ &= \mathbf{W}_B \mathbf{T}_{A\to B}^\dagger \mathbf{P}_{B\to A} + \sum_z \delta\, z\, \mathbf{W}_B \mathbf{T}_{A\to B}^\dagger \mathbf{T}_{B\to A}^\dagger\, \partial_z \mathbf{P}_B + \mathbf{R}_\perp \\ &= \mathbf{W}_{B\to A} \left\{ \mathbf{P}_{B\to A} + \sum_z \delta\, z\, \partial_z \mathbf{P}_{B\to A} \right\} + \mathbf{R}_\perp, \end{aligned}$$

where $\mathbf{W}_{B\to A} = \mathbf{W}_B \mathbf{T}_{A\to B}^\dagger$ is a new weight matrix for $\mathbf{P}_{B\to A}$ and $\partial_z \mathbf{P}_{B\to A} = \mathbf{T}_{B\to A}^\dagger (\partial_z \mathbf{P}_B)$ is the aligned manifold perturbation.

Linear Feed-forward Network (LFFN) Models

In the main text, we considered three different types of LFFN. They commonly had the movement kinematics-independent and dependent components for output variables ($\mathbf{Y}$, $\partial_\mathbf{z} \mathbf{Y}$) and input ($\mathbf{X}$, $\partial_\mathbf{z} \mathbf{X}$). Then, we compute the expectation value of the least-square error given the distribution of $\mathbf{z}$, $p(\mathbf{z})$,

$$
\begin{aligned}
E(\mathbf{T}) &= \int d\mathbf{z}\, p(\mathbf{z}) \left\| \mathbf{Y} + \sum_z \delta z\, \partial_z \mathbf{Y} - \mathbf{T}\left(\mathbf{X} + \sum_z \delta z\, \partial_z \mathbf{X}\right)\right\|^2 \\
&= \int d\mathbf{z}\, p(\mathbf{z}) \|\mathbf{Y} - \mathbf{TX}\|^2 + \int d\mathbf{z}\, p(\mathbf{z}) \sum_z \delta z\, (\partial_z \mathbf{Y}) \cdot \mathbf{TX} + (\mathbf{X} \leftrightarrow \mathbf{Y}) \\
&\quad + \int d\mathbf{z}\, p(\mathbf{z}) \sum_z \sum_{z'} \delta z \delta z' (\partial_z \mathbf{Y} - \mathbf{T}\, \partial_z \mathbf{X}) \cdot (\partial_{z'} \mathbf{Y} - \mathbf{T}\, \partial_{z'} \mathbf{X})\,.
\end{aligned}
$$

We assume that $p(\mathbf{z})$ is approximately the Gaussian distribution with zero mean. Then, we get the error function in Equation 3 in **Methods**,

$$
E(\mathbf{T}) = \|\mathbf{Y} - \mathbf{TX}\|^2 + \sum_z \sum_{z'} \mathrm{Cov}[z, z'](\partial_z \mathbf{Y} - \mathbf{T}\, \partial_z \mathbf{X}) \cdot (\partial_{z'} \mathbf{Y} - \mathbf{T}\, \partial_{z'} \mathbf{X}).
$$

In evaluating the performance, we measured the total variability by replacing the prediction ($\mathbf{TX}$, $\mathbf{T}\, \partial_z \mathbf{X}$) in Equation 3 by the mean $\langle \mathbf{Y} \rangle$ and $\langle \partial_z \mathbf{Y} \rangle$ and compared it to $E(\mathbf{T})$ to evaluate the goodness of fit, R^2. To prevent overfitting, we used the LASSO scheme[10] to minimize $E(\mathbf{T}) + \sum_{i,j} \lambda_i |T_{i,j}|$. We used the MATLAB package *glmnet*[11] and chose optimal λ_i for each Y_i by finding where AIC minimizes.

The communication subspace model (**Supplementary fig. 8d-e**) was obtained by the rank-reduced regression[12] with the error function $E(\mathbf{T})$. We first performed the unconstrained optimization of $E(\mathbf{T})$ to find the optimal least-square solution $\mathbf{T}_{\mathrm{OLS}}$. Then, we computed the d principal components of the predictor, $\mathbf{V}$, and obtained the reduced-rank solution $\mathbf{T}_{\mathrm{RRR}} = \mathbf{V}\mathbf{V}^{\dagger}\mathbf{T}_{\mathrm{OLS}}$.

Supplementary references

1 Schwartz, A. B. Motor cortical activity during drawing movements: population representation during sinusoid tracing. *J Neurophysiol* **70**, 28-36 (1993). https://doi.org:10.1152/jn.1993.70.1.28

2 Sakurai, J. J. & Napolitano, J. *Modern quantum mechanics*. Pearson new international, second edition. edn, (Pearson, 2014).

3 Greenbaum, A., Li, R.-c. & Overton, M. L. First-order perturbation theory for eigenvalues and eigenvectors. *SIAM review* **62**, 463--482 (2020).

4 Mazzucato, L., Fontanini, A. & La Camera, G. Stimuli Reduce the Dimensionality of Cortical Activity. *Front Syst Neurosci* **10**, 11 (2016). https://doi.org:10.3389/fnsys.2016.00011

5 Litwin-Kumar, A., Harris, K. D., Axel, R., Sompolinsky, H. & Abbott, L. F. Optimal Degrees of Synaptic Connectivity. *Neuron* **93**, 1153-1164.e1157 (2017). https://doi.org:10.1016/j.neuron.2017.01.030

6 Bro, R., Kjeldahl, K., Smilde, A. K. & Kiers, H. A. L. Cross-validation of component models: a critical look at current methods. *Anal Bioanal Chem* **390**, 1241-1251 (2008). https://doi.org:10.1007/s00216-007-1790-1

7 Sussillo, D., Churchland, M. M., Kaufman, M. T. & Shenoy, K. V. A neural network that finds a naturalistic solution for the production of muscle activity. *Nat Neurosci* **18**, 1025-1033 (2015). https://doi.org:10.1038/nn.4042

8 Gallego, J. A. *et al.* Cortical population activity within a preserved neural manifold underlies multiple motor behaviors. *Nat Commun* **9**, 4233 (2018). https://doi.org:10.1038/s41467-018-06560-z

9 Gallego, J. A., Perich, M. G., Chowdhury, R. H., Solla, S. A. & Miller, L. E. Long-term stability of cortical population dynamics underlying consistent behavior. *Nat Neurosci* **23**, 260-270 (2020). https://doi.org:10.1038/s41593-019-0555-4

10 Hastie, T., Tibshirani, R. & Friedman, J. H. *The elements of statistical learning : data mining, inference, and prediction*. 2nd edn, (Springer, 2009).

11 Friedman, J., Hastie, T. & Tibshirani, R. Regularization Paths for Generalized Linear Models via Coordinate Descent. *J Stat Softw* **33**, 1-22 (2010).

12 Semedo, J. D., Zandvakili, A., Machens, C. K., Byron, M. Y. & Kohn, A. Cortical areas interact through a communication subspace. *Neuron* **102**, 249-259. e244 (2019).
