# Supplementary material for: Multidimensional cerebellar computations for flexible kinematic control of movements
Source: Nat Commun. 2023 May 3;14:2548. doi: 10.1038/s41467-023-37981-0 (PMC10156706; doi:10.1038/s41467-023-37981-0)
Supplement: Supplementary file 2 — Reporting Summary [file 41467_2023_37981_MOESM2_ESM.pdf]

nature portfolio

Corresponding author(s): Peter Thier

Last updated by author(s): Apr 5, 2023

Reporting Summary

Nature Portfolio wishes to improve the reproducibility of the work that we publish. This form provides structure for consistency and transparency in reporting. For further information on Nature Portfolio policies, see our Editorial Policies and the Editorial Policy Checklist.

Statistics

For all statistical analyses, confirm that the following items are present in the figure legend, table legend, main text, or Methods section.

n/a	Confirmed	
☐	☒	The exact sample size (*n*) for each experimental group/condition, given as a discrete number and unit of measurement
☐	☒	A statement on whether measurements were taken from distinct samples or whether the same sample was measured repeatedly
☐	☒	The statistical test(s) used AND whether they are one- or two-sided<br>*Only common tests should be described solely by name; describe more complex techniques in the Methods section.*
☐	☒	A description of all covariates tested
☒	☐	A description of any assumptions or corrections, such as tests of normality and adjustment for multiple comparisons
☐	☒	A full description of the statistical parameters including central tendency (e.g. means) or other basic estimates (e.g. regression coefficient) AND variation (e.g. standard deviation) or associated estimates of uncertainty (e.g. confidence intervals)
☐	☒	For null hypothesis testing, the test statistic (e.g. *F*, *t*, *r*) with confidence intervals, effect sizes, degrees of freedom and *P* value noted<br>*Give P values as exact values whenever suitable.*
☒	☐	For Bayesian analysis, information on the choice of priors and Markov chain Monte Carlo settings
☒	☐	For hierarchical and complex designs, identification of the appropriate level for tests and full reporting of outcomes
☒	☐	Estimates of effect sizes (e.g. Cohen's *d*, Pearson's *r*), indicating how they were calculated

Our web collection on statistics for biologists contains articles on many of the points above.

Software and code

Policy information about availability of computer code

Data collection	We recorded Purkinje cell and mossy fiber activity using commercially available glass-coated tungsten microelectrodes manufactured by Alpha Omega Engineering (version3.11), Nazareth, Israel. Electrode position was controlled using a modular multielectrode manipulator (Electrode Positioning System and Multi-Channel Processor, Alpha Omega Engineering). We used Alpha Omega Engineering Multi Spike Detector (MSD), for online spike sorting. Complex spikes were detected off-line using a neural networks approach (described in Markanday et al. 2020).All experimental parameters were designed and controlled, and behavioral measures (i.e., eye movements) were recorded using in-house Linux-based software, NREC (http://nrec.neurologie.uni-tuebingen.de).
Data analysis	Data were analyzed by custom codes written in MATLAB 2020b (MathWorks, MA, USA). The data analysis codes for each main and supplementary figures are available at https://doi.org/10.5281/zenodo.7732421.

For manuscripts utilizing custom algorithms or software that are central to the research but not yet described in published literature, software must be made available to editors and reviewers. We strongly encourage code deposition in a community repository (e.g. GitHub). See the Nature Portfolio guidelines for submitting code & software for further information.

Data

Policy information about availability of data

All manuscripts must include a data availability statement. This statement should provide the following information, where applicable:

- Accession codes, unique identifiers, or web links for publicly available datasets
- A description of any restrictions on data availability
- For clinical datasets or third party data, please ensure that the statement adheres to our policy

All neural and behavioral data generated in this study is available at: https://doi.org/10.5281/zenodo.7732421

Human research participants

Policy information about studies involving human research participants and Sex and Gender in Research.

Reporting on sex and gender	*Use the terms sex (biological attribute) and gender (shaped by social and cultural circumstances) carefully in order to avoid confusing both terms. Indicate if findings apply to only one sex or gender; describe whether sex and gender were considered in study design whether sex and/or gender was determined based on self-reporting or assigned and methods used. Provide in the source data disaggregated sex and gender data where this information has been collected, and consent has been obtained for sharing of individual-level data; provide overall numbers in this Reporting Summary. Please state if this information has not been collected. Report sex- and gender-based analyses where performed, justify reasons for lack of sex- and gender-based analysis.*
Population characteristics	*Describe the covariate-relevant population characteristics of the human research participants (e.g. age, genotypic information, past and current diagnosis and treatment categories). If you filled out the behavioural & social sciences study design questions and have nothing to add here, write "See above."*
Recruitment	*Describe how participants were recruited. Outline any potential self-selection bias or other biases that may be present and how these are likely to impact results.*
Ethics oversight	

Note that full information on the approval of the study protocol must also be provided in the manuscript.

Field-specific reporting

Please select the one below that is the best fit for your research. If you are not sure, read the appropriate sections before making your selection.

☒ Life sciences ☐ Behavioural & social sciences ☐ Ecological, evolutionary & environmental sciences

For a reference copy of the document with all sections, see nature.com/documents/nr-reporting-summary-flat.pdf

Life sciences study design

All studies must disclose on these points even when the disclosure is negative.

Sample size	We collected high quality data from a large number of Purkijne cells (n=151) and mossy fibers (117) using single-unit recordings. Compared to many other studies on single-unit recordings in Purkinje cells, these numbers are much larger. Furthermore, each unit was tested in only one horizontal direction (centrifugally left or right) for an average of around 300 trials. Due to short inter-trial intervals used in our experimental paradigm, we could also use data from centripetal saccades made in the opposite direction. In short, a relatively large number of units tested for a large number of eye movement trials gave us sufficient data to gain enough confidence in our results. Therefore, we did not use any sample size determination.
Data exclusions	We excluded 4 mossy fiber units from our analysis as their responses did not fall into any of the four categories we defined. We explained those in the Method section.
Replication	Most of the times, only one Purkinje cell or mossy fiber could be tested in an individual experimental session comprising on average (median) 300 trials. The number of experimental sessions varied from 0 (if no unit could be isolated or the monkey was not motivated enough) around 4 on a single day. If , monkeys were given sufficient breaks in-between to recover.<br>In data analysis, we verified our findings in rigorous statistical analysis. For each evaluation, we performed the jackknife (leave-one-out) scheme to estimate how the finding varies when one data set is excluded. For each recording, the statistical models for predicting firing rate from behavior were fitted first to the train data, containing a half of the trials, and then tested in the test data, the rest of the trials. This procedure was performed once for each recording. See Methods for the details
Randomization	No random allocation was used since we did not use multiple conditions to compare. Data were collected and analyzed at the neuronal level with an assumption of the same condition across different animals and experimental sessions.

Blinding	No blinding was used since we did not use multiple conditions to compare.

Reporting for specific materials, systems and methods

We require information from authors about some types of materials, experimental systems and methods used in many studies. Here, indicate whether each material, system or method listed is relevant to your study. If you are not sure if a list item applies to your research, read the appropriate section before selecting a response.

Materials & experimental systems

n/a	Involved in the study
☒	☐ Antibodies
☒	☐ Eukaryotic cell lines
☒	☐ Palaeontology and archaeology
☐	☒ Animals and other organisms
☒	☐ Clinical data
☒	☐ Dual use research of concern

Methods

n/a	Involved in the study
☒	☐ ChIP-seq
☒	☐ Flow cytometry
☒	☐ MRI-based neuroimaging

Animals and other research organisms

Policy information about studies involving animals; ARRIVE guidelines recommended for reporting animal research, and Sex and Gender in Research

Laboratory animals	Two adult male rhesus macaques (Macaca mulatta) of age 10 (monkey K) and 8 (monkey E) years, purchased from the German Primate Center, Giittingen.
Wild animals	No wild animals were used in this study
Reporting on sex	We used two male rhesus macaques (Macaca mulatta; age: 10 years and 8 years, respectively) for this study. Our study focuses on computational principles embedded in distinct elements of the cerebellar circuitry for the control of eye movements. Given the vast similarity in the architecture of the cerebellum, not only within species (males or females), but also across several species, it is reasonable to assume that the underlying computational principles in the two sexes are also the same. This is why, additional female monkeys were not considered necessary for this study. Furthermore, our state's ethics board overseeing work on non-human primates research is strictly opposed to unnecessarily increasing the number of animals used in a study in order to obtain data for both sexes, unless there is a very compelling argument that sex-dependent differences can be expected – which is clearly not the case here.
Field-collected samples	No field collected sample was used in this study.
Ethics oversight	All procedures strictly adhered to the rules defined by the German as well as the European law and guidelines that were approved by the local authority (Regierungspräsidium Tübingen, veterinary license N7/18 and N4/14) and National Institutes of Health's Guide for the Care and Use of Laboratory Animals. All training, experimental and surgical procedures were supervised by the veterinary service of Tübingen University.

Note that full information on the approval of the study protocol must also be provided in the manuscript.
